# What are effective strategies to respond to the psychological impacts of working on the frontlines of a public health emergency?

**DOI:** 10.3389/fpubh.2023.1282296

**Published:** 2023-11-07

**Authors:** Sarah E. Neil-Sztramko, Emily Belita, Stephanie Hopkins, Diana Sherifali, Laura Anderson, Emma Apatu, Lydia Kapiriri, Jean Eric Tarride, Olivier Bellefleur, Sharon Kaasalainen, Sharon Marr, Maureen Dobbins

**Affiliations:** ^1^Department of Health Research Methods, Evidence, and Impact (HEI), McMaster University, Hamilton, ON, Canada; ^2^National Collaborating Centre for Methods and Tools, Hamilton, ON, Canada; ^3^School of Nursing, McMaster University, Hamilton, ON, Canada; ^4^Health, Aging & Society, McMaster University, Hamilton, ON, Canada; ^5^Center for Health Economics and Policy Analysis, McMaster University, Hamilton, ON, Canada; ^6^Programs for Assessment of Technology in Health, Research Institute of St. Joe's Hamilton, St. Joseph's Healthcare Hamilton, Hamilton, ON, Canada; ^7^National Collaborating Centre for Healthy Public Policy, Montreal, QC, Canada; ^8^Unity Health, Toronto, ON, Canada

**Keywords:** mental health, healthcare workers, COVID-19, public health emergency, education/awareness and skill development/training, prevention

## Abstract

**Background:**

The COVID-19 pandemic has disrupted the healthcare and public health sectors. The impact of working on the frontlines as a healthcare or public health professional has been well documented. Healthcare organizations must support the psychological and mental health of those responding to future public health emergencies.

**Objective:**

This systematic review aims to identify effective interventions to support healthcare workers’ mental health and wellbeing during and following a public health emergency.

**Methods:**

Eight scientific databases were searched from inception to 1 November 2022. Studies that described strategies to address the psychological impacts experienced by those responding to a public health emergency (i.e., a pandemic, epidemic, natural disaster, or mass casualty event) were eligible for inclusion. No limitations were placed based on study design, language, publication status, or publication date. Two reviewers independently screened studies, extracted data, and assessed methodological quality using the Joanna Briggs Institute critical appraisal tools. Discrepancies were resolved through discussion and a third reviewer when needed. Results were synthesized narratively due to the heterogeneity of populations and interventions. Outcomes were displayed graphically using harvest plots.

**Results:**

A total of 20,018 records were screened, with 36 unique studies included in the review, 15 randomized controlled trials, and 21 quasi-experimental studies. Results indicate that psychotherapy, psychoeducation, and mind–body interventions may reduce symptoms of anxiety, burnout, depression, and Post Traumatic Stress Disorder, with the lowest risk of bias found among psychotherapy interventions. Psychoeducation appears most promising to increase resilience, with mind–body interventions having the most substantial evidence for increases in quality of life. Few organizational interventions were identified, with highly heterogeneous components.

**Conclusion:**

Promoting healthcare workers’ mental health is essential at an individual and health system level. This review identifies several promising practices that could be used to support healthcare workers at risk of adverse mental health outcomes as they respond to future public health emergencies.

**Systematic review registration**: https://www.crd.york.ac.uk/prospero/display_record.php?RecordID=203810, identifier #CRD42020203810 (PROSPERO).

## 1 Introduction

The COVID-19 pandemic has been a significant disruption to health systems worldwide. Throughout the lifting and reinstatement of public health restrictions and waves of heightened pandemic activity, there was a growing recognition of a “shadow pandemic” of adverse mental health effects due to the pandemic (1). Healthcare workers (HCWs) in various settings are particularly at risk for significant and prolonged impacts given their frontline role in pandemic response. Previous research has demonstrated long-standing psychological consequences experienced by HCWs after SARS (2, 3), and it is expected that there will be large-scale and long-term psychological impacts due to responding to COVID-19 among several occupational groups (4–8). Several systematic reviews of studies exploring the effects of the COVID-19 pandemic on the mental health of HCWs have reported a high prevalence of anxiety, depressive symptoms, insomnia, burnout, emotional exhaustion, and somatic symptoms (9, 10). Notably, factors such as the incidence of infection in the local setting, peer infections or deaths, and a shortage of personal protective equipment all increased the likelihood of experiencing adverse mental health outcomes (9, 10). At the same time, the availability of psychological support resources had a protective effect (9). With this knowledge, an action plan, support, and resources for individuals and organizations are needed to adequately support those experiencing or at risk for psychological distress or adverse mental health outcomes.

There is currently limited guidance on how best to support HCWs in response to and following public health emergencies, such as pandemics. Recent syntheses have gathered very low- to moderate-certainty evidence about interventions that may positively affect healthcare workers’ mental health during a disease outbreak. However, these reviews have focused only on narrow categories of intervention, for example, online mindfulness programs (11), or limited populations, for example, those experiencing burnout (12). There is a need for a high-quality systematic review that captures a broad range of mental health-promoting interventions delivered during various public health emergencies to further inform strategies to improve mental health in HCWs. This review aims to answer the question: What are effective strategies to address the psychological impacts [i.e., anxiety, depression, and Post Traumatic Stress Disorder (PTSD)] experienced by HCWs responding to a public health emergency?

## 2 Methods

The protocol for this systematic review was registered (PROSPERO #CRD42020203810) and published *a priori*. There were no deviations from this protocol. The Cochrane Handbook for Systematic Reviews of Interventions guided our review protocol (13).

### 2.1 Search strategy

**Figure 1:**
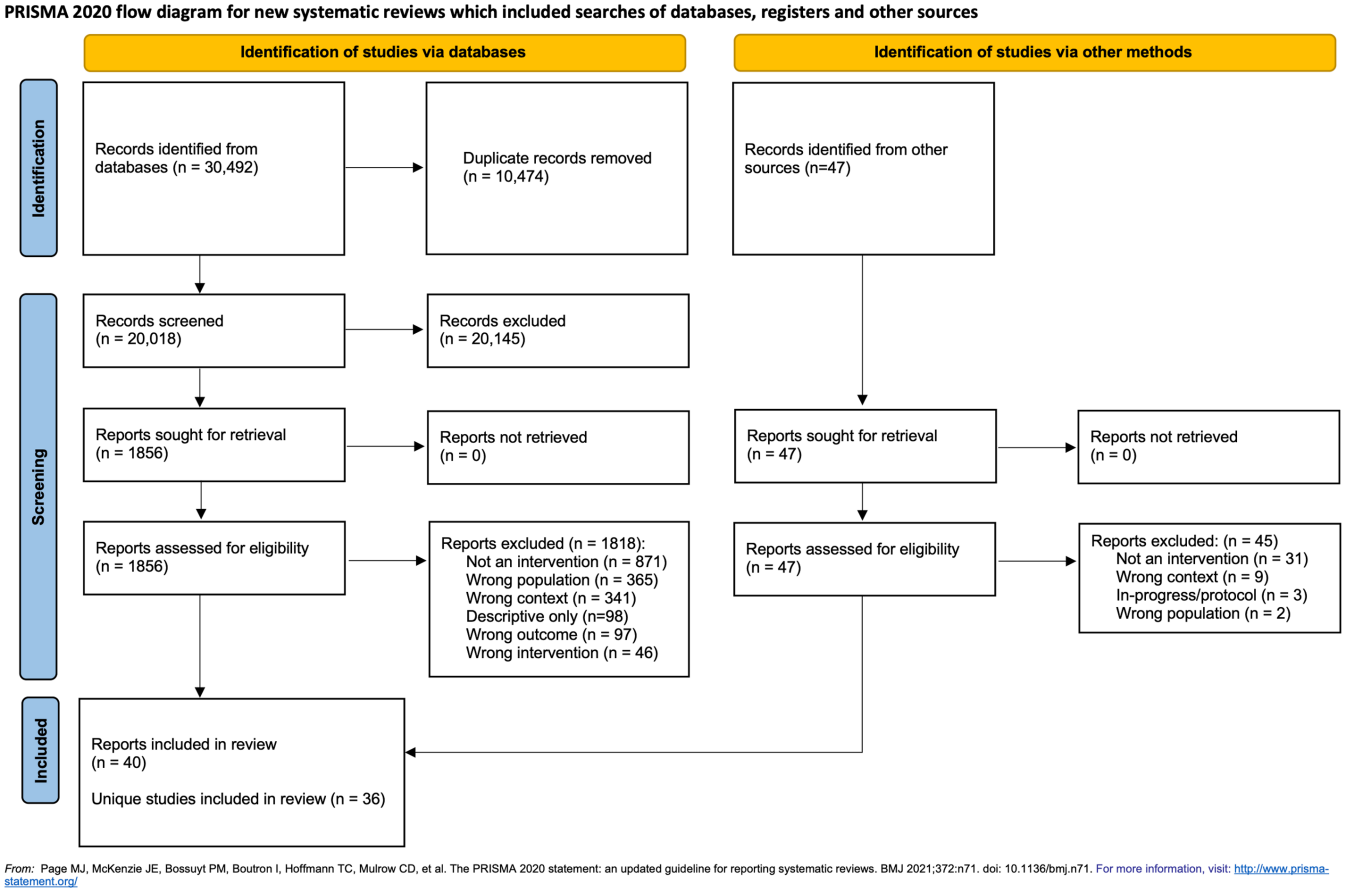
PRISMA diagram.

In collaboration with a health sciences librarian, a comprehensive search strategy was developed and executed in MEDLINE, PsycINFO, Embase, EMCARE, CINAHL, Sociological Abstracts, Business Source Premier, and the Cochrane Central Register of Controlled Trials from inception to 1 November 2022. Search terms related to (1) healthcare personnel; (2) pandemic, epidemic, COVID-19, natural disaster, or public health emergency; (3) psychological supports, interventions, and programs; and (4) mental health outcomes were used (see Supplementary Files, Figure 1 for complete MEDLINE search strategy). Reference lists of included studies and relevant systematic reviews were hand-searched, and relevant experts were contacted to identify any studies that were not captured via our search. Gray literature sources were searched until 22 January 2022, following the Canadian Agency for Drugs and Technologies in Health Grey Matters tool for searching health-related gray literature to identify unpublished studies (14).

### 2.2 Inclusion and exclusion criteria

Eligible studies described interventions to prevent, minimize, or treat adverse psychological or mental health or wellbeing at an individual, organizational, or systemic level resulting from a public health emergency, defined as emergent situations as those in which the scale, timing, and unpredictability of health consequences threaten to overwhelm routine capabilities (15); for our purposes, we defined these as an epidemic, pandemic, natural disaster, or mass casualty event. The population was limited to frontline HCWs who were involved in the response to the public health emergency. Ongoing public health crises without distinct starting points are excluded from this contextual requirement (e.g., the opioid epidemic, poverty, homelessness as epidemics, or the prolonged HIV/AIDS epidemic). HCWs included medically trained healthcare professionals typically considered at the frontlines (e.g., nurses and physicians in acute care, outpatient, public health, and long-term care) and allied health professions (e.g., personal support workers, infection control professionals, and social workers). Emergency and law enforcement workers whose work is not primarily associated with health (e.g., police and firefighters; non-health-related essential workers such as grocery store employees and cleaning staff; and civilian volunteer emergency responders) were excluded. Any comparison group, such as placebo control, standard care, or pre-intervention data, was included. Studies that reported occupational, psychological, or mental health outcomes were all included. For this review, mental health is defined per the World Health Organization’s definition as “a state of mental wellbeing that enables people to cope with the stresses of life, realize their abilities, learn well and work well, and contribute to their community” (16). These outcomes were defined *a priori* by the research team as anxiety, depression, burnout, exhaustion, resilience, altered mood, or clinical diagnoses of mental disorders such as depression or PTSD. Descriptive-only or in-progress studies that described interventions without reporting on mental health or wellbeing outcomes were excluded. No restrictions were placed on study design, language, publication status, publication date, or study location.

### 2.3 Study selection and data extraction

Once databases were searched and duplicates were removed, the title and abstract of each reference were screened independently by two trained reviewers. Full texts of all potentially relevant articles were retrieved and screened independently by two experienced reviewers. Disagreements were resolved through discussion or a third team member when needed.

Two trained team members independently completed data extraction and critical appraisal, with discrepancies resolved through discussion or a third team member when needed. A data extraction form was created and trialed with three studies and contained 42 items related to study characteristics (study aim, study design, and country); study sample (participant age, gender, professional role, organization, and sample size); intervention and comparator groups; and outcomes (primary and secondary outcomes, measurement methods including anxiety; depression; PTSD; burnout; quality of life; resilience and coping; and mood-related outcomes). Mean differences between groups were extracted to compare outcomes across studies with expected results. Where within-group baseline and end-of-study data were reported, they were transformed into the mean difference and standard deviation of the difference using guidance from the Cochrane Handbook for Systematic Reviews (17). The intervention-related information was extracted using the template for intervention description and replication (TIDieR) checklist (18). Critical appraisal was completed using the appropriate Joanna Briggs Institute critical appraisal tool for the study design extracted (19). Studies rated as unclear or high risk of bias on more than half of domains were defined as having an increased risk of bias; those rated as unclear or high for three or more domains were defined as having some concerns.

### 2.4 Data synthesis

A visual examination of the data extraction table organized by contextual information (i.e., disaster type, setting, and intervention) revealed significant heterogeneity in the data; thus, a meta-analysis was deemed inappropriate. Interventions were categorized post-hoc as falling within four broad categories to support the narrative synthesis: psychotherapy, psychoeducation, mind–body, and organizational interventions; studies that did not fit within one of these categories were classified as “other.” Harvest plots were created for each intervention type and mode of delivery to display study outcomes graphically. Each row of the plot showed effect sizes for each category of results extracted (e.g., anxiety, depression, PTSD, burnout, quality of life, resilience or coping, and mood), with the size of the bar indicating the effect size. Effect size estimates were extracted from the published articles when reported as Cohen’s d or Hedge’s g or were calculated using the Carlson–Schmidt method for paired pre-treatment and post-treatment data (20, 21) or the Campbell Collaboration’s effect size calculator created by Wilson (22, 23). Bars were color-coded by study design (RCT vs. quasi-experimental) and risk of bias (low, some concerns, and high), and studies with a statistically significant change were labeled.

## 3 Results

### 3.1 Characteristics of included studies

Across databases, 30,492 records were identified, along with 47 from reference lists and gray literature (Figure 1). Following de-duplication, the remaining 20,018 records were screened by title and abstract. Of the 1,856 articles assessed at the full-text level, 39 publications describing 36 unique interventions met eligibility criteria (Figure 1) (24–62).

Studies were published between 1994 and 2022 (Table 1). Nine studies were conducted in the United States (26, 29, 38, 39, 41, 42, 44, 50, 56), three in the United Kingdom (25, 36, 59), China (27, 34, 52), Italy (40, 60, 61), and Taiwan (28, 37, 58), Turkey (46, 54, 57), and two in Sierra Leone (24, 53). The remaining 10 studies were conducted in Armenia (37), Chile (30), Indonesia (48), Iran (62), Israel (45), Japan (49) and the Philippines (55), Singapore (51), South Africa (31), and Spain (35). Interventions were delivered in response to an infectious disease pandemic or epidemic (*n* = 24, 67%), namely COVID-19 (*n* = 20, 56%) (25, 27, 31, 34–36, 40, 44, 46–48, 50–52, 54, 57, 59–62), Ebola (*n* = 3, 8%) (24, 29, 53), and SARS (*n* = 1, 2%); natural disasters (*n* = 10, 38%), namely earthquakes (*n* = 4, 11%) (28, 30, 37, 49), hurricanes (*n* = 5, 14%) (38, 39, 41, 42, 56), and typhoons (*n* = 1, 3%) (55); or mass casualty events (*n* = 2, 6%) (26, 45).

**Table 1:** Description of interventions.

Author, Year	Disaster, Year	Setting	Study Design	N	Participants	Age (Mean, (SD))	Sex (% F)	Intervention	Comparator	Duration	Follow-Up
Psychotherapy interventions (*n* = 9)											
Cole (24, 32, 33)	Ebola, 2013–2015	Ebola treatment centers, Sierra Leone	Single-group pre–post	253	Former Ebola treatment center staff	30 (7.0)	23 (6% NR)	Group CBT plus home booklet, single 3 h session/week	Pre-intervention	6 weeks	2 weeks
De Kock (25)	COVID-19	NHS Highland, UK	RCT	107	Health or social care workers in NHS	18–25: 2.4%; 26–30: 5.9%; 31–40: (18.3); >40: 73.4	88	My Personal Self app, evidence-based app using CBT and positive psychology, adapted for COVID-19 context (narrative of delivery, text message prompts, 24-h support line)	CG1: WL; CG2: original My Personal Self app	4 weeks	Immediate
Difede (26)	11 September Attacks, 2001	New York City, USA	RCT	21	Disaster relief workers	45.8 (7.7)	3	In-person CBT by a licensed psychologist (75 min session/week) plus daily homework	Usual care	12 weeks	Immediate and 3 months later
Jing (27)	COVID-19	Tertiary general hospitals, Xuzhou City, China	Single-group pre–post	226	Nurses	≤30: 61.9%; 31–40: 29.6%; 41–50: 7.1%; 51–60: 1.3%	95	Group-based ACT program; two modules focused on work and life; participants encouraged to participate in mindfulness daily	Pre-intervention	10 weeks	Immediate
Ke (28)	Taiwan Earthquake, 2016	Chi-Mei Medical Center, Tainan City, Taiwan	Single-group pre–post	67	HCW, physicians, and nurses	32.7 (5.2)	53	Single session, in-person PFA, debriefing, and minilectures. Physical therapists provided muscle and mental relaxation programs.	Pre-intervention	1 session	1 month later
Klomp (29)	Ebola, 2014–2016	CDC deployment, USA	Single-group pre–post	~100	Public health staff	NR	NR	Deployment Safety and Resilience Team training based on PFA	Pre-intervention	Variable	Immediate
Leiva-Bianchi (30)	Chile Earthquake and Tsunami, 2010	Hospital, public department, and school, Constituçion and Talca, Chile	Non-randomized intervention	29	HCW, public department workers, and teachers with 3+ severe symptoms of PTSD	IG: 46 (NR) CG1: 48 (NR) CG2: 51 (NR)	IG: 100 CG1: 88 CG2: 75	In-person 60–90 min weekly group CBT session led by trained therapists and included psychoeducation, breathing retraining, behavioral activation, and cognitive restructuring	CG1: Individuals without PTSD CG2: Abbreviated intervention	10–12 weeks	Immediate
Osman (31)	COVID-19	South Africa	Single-group pre–post	47	HCW	Median: 34, IQR: 18	NR	Mindfulness-based stress reduction program delivered by a clinical psychologist	Pre-intervention	4 weeks	Immediate
Zhou (34)	COVID-19	Tianjin Medical University Hospital Airport Site, China	RCT	118	Nurses with insomnia	29.6 (4.5)	98	e-CBT for insomnia	No intervention	6 weeks	Immediate
Psychoeducation interventions (*n* = 11)											
Berger (45)	Lebanon War, 2006	Primary Care Clinic, Israel	RCT	80	Well baby clinic nurses	IG: 49.3 (7.4) CG: 47.7 (7.1)	100	6-h weekly group-based psychoeducational sessions to improve knowledge and self-management including tools such as breathing, meditation, relaxation, exercise, self-affirmation, and guided imagery.	Wait-list control	12 weeks	3 months
Fiol-DeRoque (35, 43)	COVID-19	Spain	RCT	482	HCW	41.4 (10.4)	83	PsyCovidApp, self-management, psychoeducational based on CBT and mindfulness including content on emotional skills, healthy lifestyle, work stress and burnout, and social support. Daily self-monitoring prompts with tailored self-management	Sham control	2 weeks	Immediate
Gnanapragasam (36)	COVID-19	NHS Trusts, England	RCT	894	NHS affiliated staff	18–29: 13.4%; 30–39: 21.1%; 40–49: 29.1%; 50–59: 26.5%; ≥60: 9.8%	84	Foundations App, to promote behavior change, positive habits, promote mental wellbeing, manage stress and improve sleep; users choose one of the six focus areas (relaxation, sleep, anxiety, feeling down, stress, self-esteem)	Usual care	8 weeks	Immediate
Karakashian (37)	Armenian Earthquake, 1988	City of Giumri, Armenia	Single-group pre–post	25	School counselors that provided psychological intervention	30–53	100	Single-session didactic-experiential group therapy to discuss experiences, coping strategies, issues, and treating trauma.	Pre-intervention	2-h seminar	Immediate
Leitch (38)	Hurricanes Katrina and Rita, 2005	New Orleans and Baton Rouge, USA	Non-randomized intervention	142	Social workers	22–39: 32.4% 40–54: 38.8% 55+: 28.8%	86	Group psychoeducation ‘Somatic ExperiencingÒ/ Trauma Resiliency ModelÔ’ plus 1–2 individual sessions with trained clinicians.	Staff who declined participation	1–2 weeks	3–4 months
Mahaffey (39)	Hurricane Sandy, 2012	Community Organization, USA	RCT	167	Disaster responders with active responder status	IG: 51.8 (15.3) CG: 51.2 (15.5)	IG: 47 CG: 59	In-person group disaster work resilience training program focused on psychoeducation to build resilience reduce stress and enhance coping strategies.	Waitlist control group	4-h session	3 months
Pallavicini (40)	COVID-19	Hospitals, Milan, Italy	Single-group pre–post	20	HCW	43 (10)	60	Virtual reality-based psychoeducational experience to reduce stress and anxiety, MIND-VR.	Pre-intervention	Single session	Immediate
Powell (41)	Hurricane Sandy, 2012	Federally qualified health centers, USA	Single-group pre–post + qualitative	69	HCW, disaster response, and social service providers	NR	80	In-person group ‘Resilience and Coping for the Healthcare Community’ workshop using a solution-focused approach	Pre-intervention	3-h workshop	Immediate and 3 weeks
Powell (42)	Hurricane Sandy, 2012	Social service organizations, New York and New Jersey, USA	Single-group pre–post	839	Social service providers, including social workers and counselors	NR	84	In-person group psychoeducational workshop, “Caregivers Journey of Hope”	Pre-intervention	Half-day workshop	Immediate
Yi-Frazier (44)	COVID-19	Children’s hospital and medical center, Seattle, USA	Single-group pre–post	153	HCW and staff,	40.6 (10.1)	92	Promoting Resilience in Stress Management (PRISM); manualized, skills-based coaching program to promote resilience and stress management	Pre–post	6 weeks	Immediate
Mind–body interventions (*n* = 12)											
Hosseinzadeh (46)	COVID-19	Ankara, Turkey	RCT	49	Social workers	33.0 (6.0)	55	Mindfulness training sessions plus daily meditation homework	Waitlist	4 weeks	1 month
Hsieh, 2022 (47)	COVID-19	Hospitals, Taiwan	RCT	79	Nurses	IG: 42.3 (8.5); CG: 32.5 (8.2)	NR	Gong meditation; Guided meditation through a series of real-time adjusted gong strikes	Smartwatch to measure stress	2 days	Immediate
Ibrahim (48)	COVID-19	Hospitals, West Java, Indonesia	Non-randomized intervention	50	Nurses	21–30: 44%; 31–40: 38%; 41–50: 18%	48	Mindfulness breathing meditation delivered via WhatsApp; included protocol of mindfulness breathing with video practice guidelines and tutorials and reflective exercises	“Control” not described	4 weeks	Immediate
Iwakuma (49)	Great East Japan Earthquake, 2011	Tohoku Region, Japan	Single-group pre–post	17	HCW	50 (NR)	94	In-person group breathing-based meditation seminar	Pre-intervention	45 min	Immediate
Joshi (50)	COVID-19	Duke Medical Centre, Durham, USA	RCT	80	HCW	40 (11)	83	Transcendental Meditation, a practice in which individuals silently recite a single mantra without concentration or contemplation. Eight instructional sessions plus 20 min, 2x/day of self-practice	Access to wellness resources	3 months	Immediate
Keng (51)	COVID-19	Duke-NUS medical school, Singapore	RCT	80	HCW	30.2 (6.2)	90	Headspace meditation app	Lumosity app	3 weeks	Immediate
Li (52)	COVID-19	Had returned from Wuhan, China	RCT	134	HCW	21–30: 34.3%; 31–40: 42.6%; 41–50: 17.1%; 51–60: N6%	70	Brief mindfulness meditation	“Control” not described	16 days	Immediate
Liu (53)	Ebola, 2013	Ebola clinic, Sierra Leone	Non-randomized intervention	41	International HCWs from China	35 (range: 24–46)	49	In-person Ba Duan Jin, a medium-to-low intensity aerobic exercise.	No intervention control	30 min daily	6 weeks
Si (54)	COVID-19	Hospital, Turkey	RCT	101	Nurses	IG: 28.9 (6.8); CG: 28.9 (5.6)	81	Laughter yoga: Stretch-relaxation, laughter, deep breathing exercises. Techniques to break down barriers to laughter and develop feelings of childish play	“Control” (not described)	4 weeks	Immediate
Waelde (56)	Hurricane Katrina, 2005	Mental Health Agency, New Orleans, USA	Single-group pre–post	20	Mental HCW and staff	49 (11)	85	In-person 4-h ‘Inner Resources for Stress’ meditation retreat and home study program 30 min/day, 6 days/week	Pre-intervention	8 weeks	3 and 8 weeks
Waelde (55)	Typhoon Haiyan, 2013	Quezon City, Philippines	Single-group pre–post	68	Mental health professionals, faculty, and students	37.3 (11.6)	75	In-person 4-h ‘Inner Resources for Stress’ meditation workshop and home study program 30 min/day, 6 days/week	Pre-intervention	8 weeks	8 weeks
Yildirim (57)	COVID-19	COVID-19 unit, University hospital, Istanbul, Turkey	RCT	104	Nurses	IG: 27.6 (5.2); CG: 29.1 (6.6)	80	Mindfulness-based breathing and music therapy, 30-min per day led by a certified therapist	Relaxed in a quiet and calm setting for 30 min	Single session	Immediate
Workplace-based interventions (*n* = 3)											
Chen (58)	SARS, 2003	SARS treatment hospital, Taiwan	Single-group pre–post	116	Nurses	31 (10.8)	98	In-service training on infection control, manpower allocation, PPE, mental health support team.	Pre-intervention	3 months	1 month
Saqib (59)	COVID-19, 2020	NHS, UK	Single-group pre–post	21	NHS staff	NR	NR	Hospital-based workplace hub for staff to destress and recuperate with board games, mindfulness activities, books, self-guided meditation, and volunteer-led yoga and mindfulness sessions.	Pre-intervention	1 month	Immediate
Zaghini (60)	COVID-19	University Hospital, Italy	Single-group, post-test	322	Nurses	43.4 (8.3)	76	Proactive management, reorganized care settings, increased staffing levels, online education to increase competence and knowledge related to COVID-19; participatory approach to intervention, unit-level focus, enhanced surveillance for COVID-19 exposed	None	NR	5 months
Other interventions (*n* = 2)											
Giordano (61)	COVID-19, 2020	University Hospital of Bari, Italy	Single-group pre–post	29	HCW ≥ 18 years old working on COVID-19 unit	31.8 (8.3)	65	Trained music therapists developed 15–20-min playlists and listening guides targeting relaxation and reducing anxiety and stress; recover energy and support concentration; and/or release tension and instill calm.	Pre-intervention	5 weeks	Immediate after listening
Mahdood (62)	COVID-19	University hospitals, Hamadan, Iran	RCT	80	HCW 18–50y, with anxiety and insomnia	IG: 31.5 (7.8); CG: 33.1 (7.6)	73	Damask Rose aromatherapy before work shift and during sleep	Paraffin oil	30 days	Immediate

CBT, cognitive behavioral therapy; CDC, Centers for Disease Control and Prevention; CG, control group; COVID-19: Coronavirus Disease 2019; EAP, employee assistance program; HCW, healthcare worker; IG, intervention group; ICU, intensive care unit; NHS, National Health Service; NR, not reported; PFA, psychological first aid; PTSD, post-traumatic stress disorder; RCT, randomized controlled trial; SARS, Severe Acute Respiratory Syndrome; UK, United Kingdom; USA, United States of America.

Fifteen studies (42%) were randomized controlled trials (25, 26, 34–36, 39, 45–47, 50–52, 54, 57, 62), 17 (47%) were single-group pre–post quantitative studies (24, 27–29, 31, 37, 40–42, 44, 49, 55, 56, 58–61), and four (11%) were non-randomized interventions with a comparator group (30, 38, 48, 53). Interventions were categorized into four intervention types: psychotherapy interventions (*n* = 9, 25%) defined as comprehensive psychological support including cognitive behavioral therapy (CBT) or training in psychological first aid (PFA), usually delivered or supported by a mental health professional (24–34); psychoeducation interventions (*n* = 11, 31%) which consisted of primarily didactic or interactive educational strategies to promote the use of self-management techniques and build resilience and coping ability (35–45); mind–body interventions (*n* = 12, 33%), such as meditation, yoga, and exercise (46–57); and workplace-based interventions (*n* = 3, 8%) such as in-service-training and workplace hubs (58–60). Two identified interventions using music therapy (61), and aromatherapy (62) did not fit well within other intervention categories and are thus presented separately as ‘other’. Most studies (*n* = 22, 61%) were delivered to a diverse group of HCWs (24, 26, 28, 30, 31, 35, 36, 39–42, 44, 49–53, 56, 59, 61, 62). In contrast, others specifically targeted nurses (*n* = 9, 25%) (27, 34, 45, 47, 48, 54, 57, 58, 60), social workers (*n* = 6) (38, 46), counselors (*n* = 1, 3%) (37), mental health professionals (*n* = 1, 3%) (55), and public health workers (*n* = 1, 3%) (29). Complete details on each intervention following the TIDieR framework can be found in the Supplementary Files, Table 1.

### 3.2 Risk of bias

**Figure 2:**
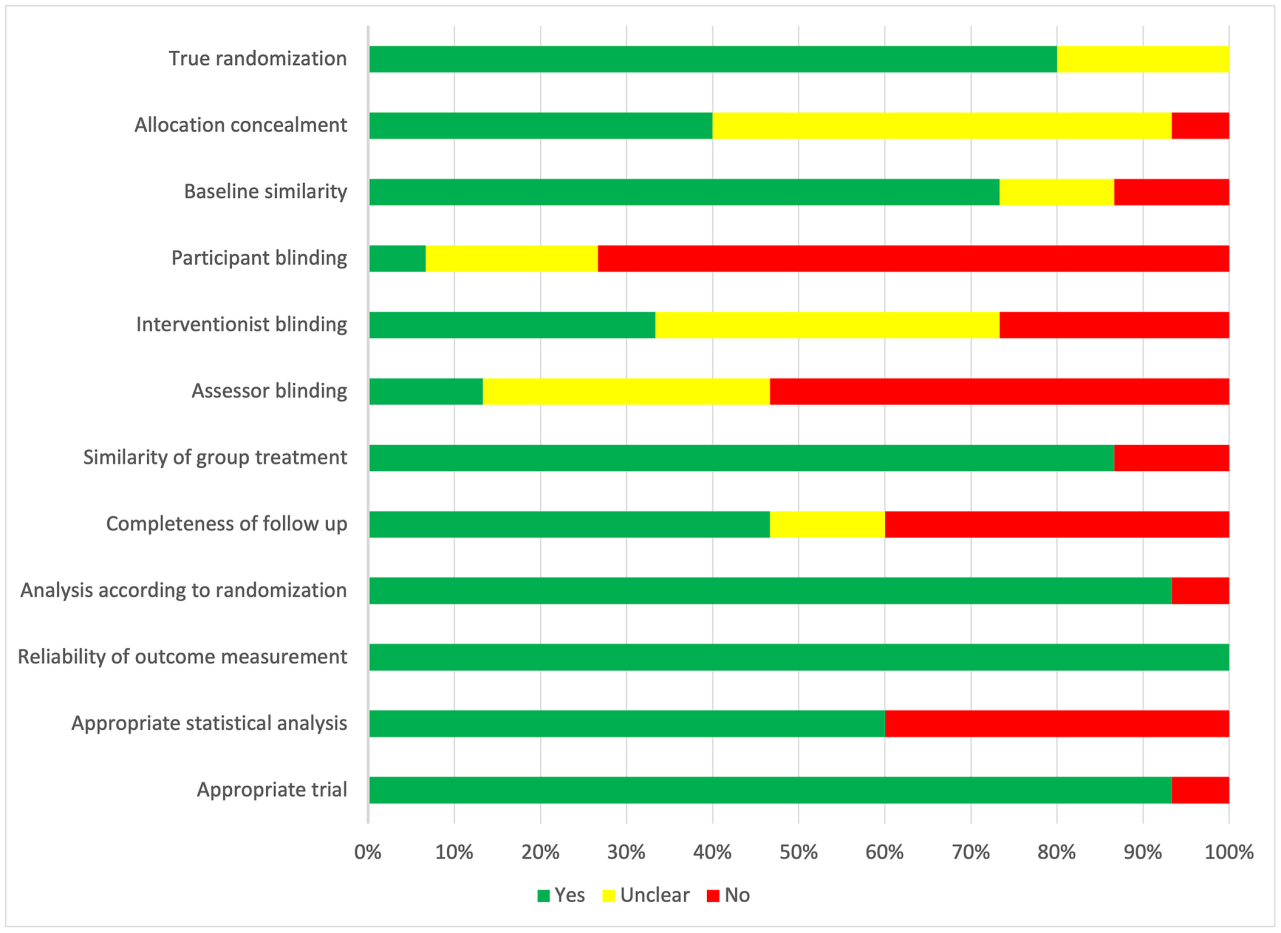
Risk of bias in randomized controlled trials.

**Figure 3:**
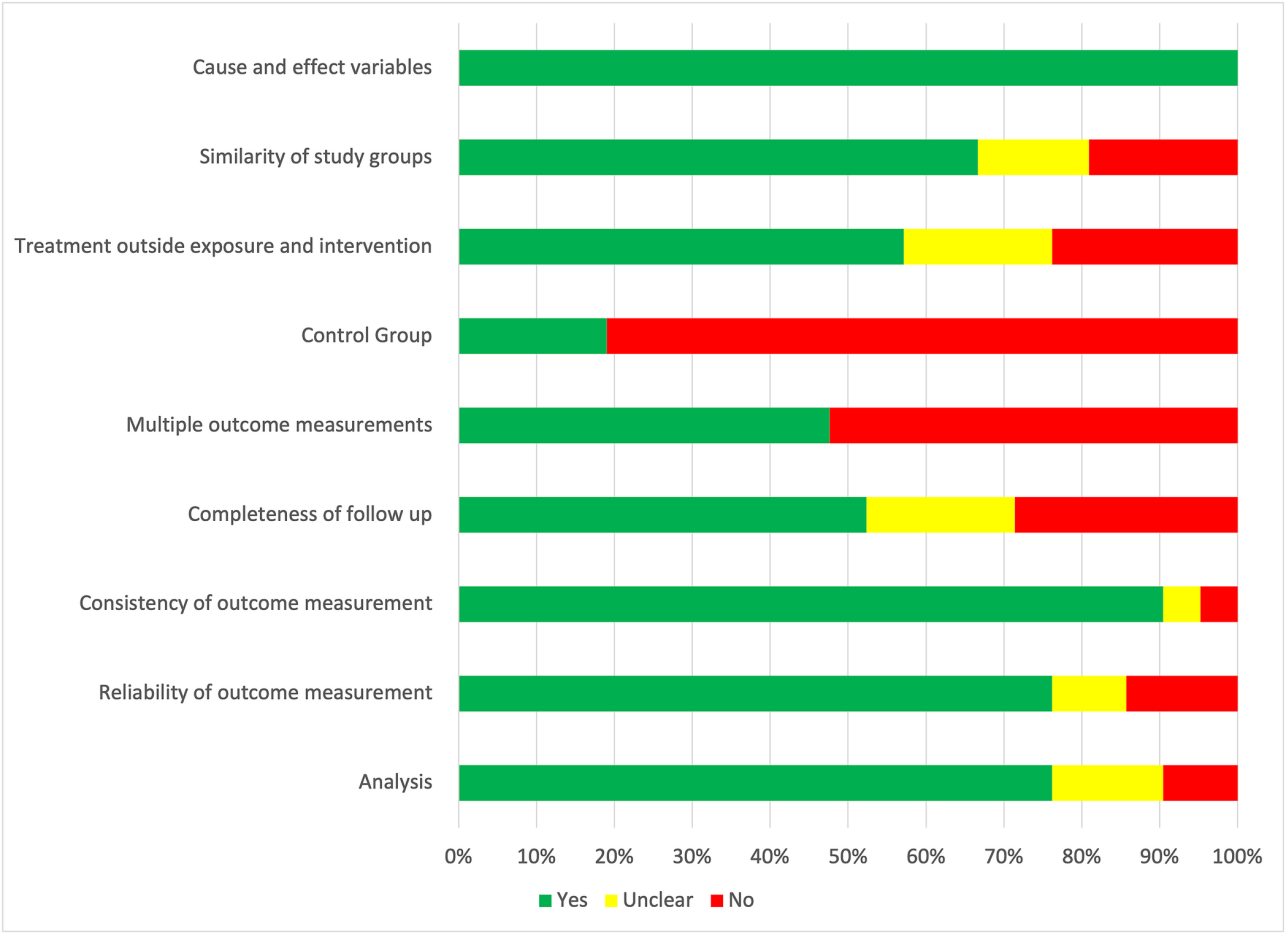
Risk of bias in quasi-experimental studies.

The risk of bias was moderate to high in most studies; within the RCTs, one study (7%) was defined as having a low risk of bias (35), nine (60%) as having some risk of bias concerns (25, 26, 36, 45, 47, 50, 51, 57, 62), and five (33%) as having a high risk of bias (34, 39, 46, 52, 54) (Figure 2). Of quasi-experimental studies, seven (33%) were rated as having a lower risk of bias (24, 27, 31, 40, 44, 58, 60), seven (33%) were rated as having some concerns (30, 38, 41, 48, 53, 59, 61), and seven (33%) as having a high risk of bias (28, 29, 37, 42, 49, 55, 56) (Figure 3). The most problematic domains within the RCTs were lack of blinding (participant, interventionist, and assessor), allocation not concealed, appropriateness of the statistical analysis, and incomplete follow-up. The most problematic domains within quasi-experimental studies were lack of a comparator group, incomplete follow-up, and the use of multiple assessment outcome measures. Full critical appraisal results can be found in Supplementary Files, Table 2.

**Table 2:** Results.

Study	Risk of bias	Tool	Results (mean difference ± SD or 95% CI, unless otherwise reported)
**Anxiety**			
**Psychotherapy**			
Cole (24, 32, 33)	Low	GAD-7 (*Phase 2*)	**−4.46 ± 4.08, *p* < 0.001**
		GAD-7 (*Phase 3*)	**−3.12 ± 4.06, *p* < 0.05**
De Kock (25)	Some concerns	GAD-7 (vs. *original app*)	Cohen’s d: +0.01 (−0.26, 0.28)
		GAD-7 (vs. *waitlist*)	Cohen’s d: −0.06 (−0.31, 0.19)
Jing (27)	Low	SCL-90 – Anxiety	**−0.20 (−0.31, −0.10)**
Zhou (34)	High	GAD-7	**INT: – 3.0 ± 3.0; CON: −0.5 ± 3.4, *p* < 0.05**
**Psychoeducation interventions**			
Fiol-DeRoque (35)	Low	DASS-21 – Anxiety	SMD: −0.04 (−0.12, 0.04)
Gnanapragasam (36)	Some concerns	GAD-7	OR: 0.70 (0.36, 1.35)
Pallavicini (40)	Low	STAI	**−4.0 ± 5.2, *p* = 0.016**
Yi-Frazier (44)	Low	GAD-7	**−2.04 (−2.74, −1.34)**
**Mind–body interventions**			
Hosseinzadeh (46)	High	DASS-21 – Anxiety subscale (*End of study*)	INT: −1.9 ± 2.7; CON: −0.9 ± 2.9, *p* = 0.17
		DASS-21 – Anxiety subscale (*Follow-up*)	INT: −1.1 ± 2.6; CON: −0.2 ± 2.9, *p* = 0.10
Joshi (50)	Some concerns	GAD-7	**−2.2 (−3.8, −0.5)**
Keng (51)	Some concerns	DASS-21 (*T2*)	−0.126 (SD: NR), *p* = 0.148
		DASS-21 (*T3*)	−0.046 (SD: NR), *p* = 0.638
Li (52)	High	GAD-7	INT: −0.52 ± 2.19; CON: – 0.85 ± 2.20, *p* > 0.05
Liu (53)	Some concerns	% self-reporting anxiety symptoms (tool NR)	−13% (SD: NR), *p* > 0.05
Waelde (56)	High	STAI	**−8.65 ± 9.0, *p* < 0.05**
Waelde (55)	High	Anxiety severity (single item, 1, not at all, to 5, extremely anxious)	−0.85 ± 0.46, p = NR
Yildirim (57)	Some concerns	STAI	**INT: −8.96 ± 9.58; CON: −0.92, 8.87, *p* = 0.01**
**Workplace-based interventions**			
Chen (58)	Low	Zung’s Self-rating Anxiety Scale	**−0.42 (−0.54, −0.29)**
**Other interventions**			
Mahdood (62)	Some concerns	STAI (*T2*)	**INT: −4.33 (0.39); CON: 0.60 (0.39), *p* < 0.001**
		STAI (*T3*)	**INT: −6.71 (0.55); CON: −1.28 (0.55), *p* < 0.001**
**Depression**			
**Psychotherapy**			
Cole (24, 32, 33)	Low	PHQ-9 (*Phase 2*)	**−4.51 ± 6.37, *p* = 0.033**
		PHQ-9 (*Phase 3*)	**−2.72 ± 5.06, *p* < 0.05**
De Kock (25)	Some concerns	PHQ-9 (vs. *original app*)	Cohen’s d: 0.19 (−0.12, 0.50)
		PHQ-9 (vs. *waitlist*)	Cohen’s d: −0.18 (−0.46, 0.11)
Difede (26)	Some concerns	BDI	INT: −2.27 ± 5.26; CON – 0.08 ± 5.29, *p* > 0.05
Jing (27)	Low	SCL-90 – Depression	**−0.30 (−0.42, −0.18)**
Zhou (34)	High	PHQ-9	INT: −3.5 ± 3.1, CON: −0.9 ± 3.1, p > 0.05
**Psychoeducation interventions**			
Fiol-DeRoque (35)	Low	DASS-21 Depression	SMD: 0.00 (−0.07, 0.08)
Gnanapragasam (36)	Some concerns	PHQ-9	OR: 0.70 (0.39, 1.28)
Karakashian (37)	High	Hamilton Rating Scale for Depression	**Significant difference, data NR**
Mahaffey (39)	High	PHQ-9	−1.2 (−2.4, 0.0)
**Mind–body interventions**			
Hosseinzadeh (46)	High	DASS-21 – Depression (*End of study*)	**INT: −3.1 ± 5.0; CON: −0.29 ± 5.5, *p* = 0.008**
		DASS-21 – Depression (*Follow-up*)	**INT: −1.4 ± 5.0; CON: +1.3 ± 5.2, *p* = 0.002**
Joshi (50)	Some concerns	PHQ-9	−1.9 (−3.7, 0.1)
Keng (51)	Some concerns	DASS-21 (*T2*)	−0.166 (SD: NR), *p* = 0.057
		DASS-21 (*T3*)	**−0.222 (SD: NR), *p* = 0.021**
Li (52)	High	PHQ-9	INT: −1.24 ± 2.88; CON: −1.46 ± 3.04, *p* > 0.05
Waelde (56)	High	CES-D	−1.68 ± 5.1, *p* > 0.05
Waelde (55)	High	CES-D	+0.10 ± 0.44, *p* = NR
**Workplace-based interventions**			
Chen, 2006 (58)	Low	Zung’s Self-rating Depression Scale	**−0.48 (−0.63, −0.33)**
**PTSD**			
**Psychotherapy**			
Cole (24, 32, 33)	Low	PCL-C (*Phase 3*)	**−12.98 ± 11.93, *p* < 0.01**
Difede (26)	Some concerns	CAPS	INT: – 11.6 ± 15.7; CON: −2.1 ± 13.4, *p* > 0.05
		PCL-C	INT: −7.7 ± 9.4; CON: +10 ± 8.0, *p* > 0.05
Ke (28)	High	Symptoms of PTSD (HCP-administered questionnaire)	**−16.4% (SD: NR), *p* < 0.05**
Leiva-Bianchi (30)	Some concerns	SPRINT-E (*with PTSD*)	**−6.0 ± 1.4, *p* < 0.01**
		SPRINT-E *(no PTSD*)	0.5 ± 1.1, *p* > 0.05
		SPRINT-E (*Abbreviated intervention with PTSD*)	−1.3 ± 2.1, *p* > 0.05
**Psychoeducation interventions**			
Fiol-DeRoque (35)	Low	DTS	SMD: 0.00 (−0.06, 0.07)
Karakashian (37)	High	DSM-III-Revised criteria for PTSD	**Significant difference, data NR**
Leitch (38)	Some concerns	PCL-C	**−6.6 (−10.5, −2.7)**
Mahaffey (39)	High	CAPS for DSM-5	−1.4 (−3.4, 0.6)
**Mind–body interventions**			
Keng (51)	Some concerns	PCL-C (*T2*)	−0.092 (SD: NR), *p* = 0.336
		PCL-C (*T3*)	−0.153 (SD: NR), *p* = 0.079
Waelde (56)	High	PCL-S	**−6.03 ± 6.24, *p* < 0.01**
**Burnout**			
**Psychotherapy**			
Osman (31)	Low	MBI (*emotional exhaustion*)	**−1.38** ± **4.6, *p* = 0.04**
		MBI (*professional accomplishment*)	**Median difference: 1, IQR 0, 3, p = 0.002**
		MBI (*depersonalization*)	Median difference: 0, IQR −2, 0, *p* = 0.15
**Psychoeducation interventions**			
Berger (45)	Some concerns	ProQOL – burnout	**−10.1 (−13.2, −6.9)**
Fiol-DeRoque (35)	Low	MBI (*emotional exhaustion*)	SMD: +0.01 (−0.06, 0.08)
		MBI (*professional accomplishment*)	SMD: −0.05 (−0.12, 0.03)
		MBI (*depersonalization*)	SMD: +0.01 (−0.06, 0.09)
Powell (41)	Some concerns	ProQOL – Burnout	−0.22 ± 2.12, *p* > 0.05
Yi-Frazier (44)	Low	MBI (*emotional exhaustion*)	**−0.37 (−0.56, −0.18)**
		MBI (*cynicism*)	**−0.22 (−0.41, −0.03)**
		MBI (*professional efficacy*)	−0.09 (−0.04, 0.23)
**Mind–body interventions**			
Hsieh (47)	Some concerns	Occupational Burnout Inventory (*Personal burnout*)	**−6.53 (SE = 1.26), *p* < 0.001**
		Occupational Burnout Inventory (*Work-related burnout*)	**−5.85 (SE = 1.37), *p* < 0.001**
		Occupational Burnout Inventory (*Client-related burnout*)	**−3.98 (SE = 1.69), *p* = 0.019**
		Occupational Burnout Inventory (*Overcommitment to work*)	**−3.69 (SE = 1.49), *p* = 0.013**
Joshi (50)	Some concerns	MBI (*emotional exhaustion*)	**−5.4 (−9.2, −1.6)**
		MBI (*professional accomplishment*)	+1.9 (−0.4, 4.1)
		MBI (*depersonalization*)	−1.7 (−3.6, 0.2)
Keng (51)	Some concerns	ProQOL – burnout (*T2*)	−0.077 (SD: NR), *p* = 0.308
		ProQOL – burnout (*T3*)	−0.127 (SD: NR), *p* = 0.098
Si (54)	High	MBI	**INT: −11.0 ± 8.0; CON: −0.4 ± 6.4, *p* < 0.001**
**Quality of life**			
**Psychotherapy**			
De Kock (25)	Some concerns	Warwick-Edinburgh Mental Wellbeing Scale (vs. *original app*)	Cohen’s d: −0.20 (−0.48, 0.08)
		Warwick-Edinburgh Mental Wellbeing Scale (vs. *waitlist*)	Cohen’s d: 0.15 (−0.10, 0.41)
**Psychoeducation**			
Berger (45)	Some concerns	ProQOL – compassion satisfaction	**+11.4 (8.4, 14.4)**
		ProQOL – compassion fatigue	**−7.6 (−10.7, −4.6)**
Gnanapragasam (36)	Some concerns	GHQ-12	**+1.34 (0.53, 2.15)**
Powell (41)	Some concerns	ProQOL – compassion satisfaction	+0.67 ± 3.58, *p* > 0.05
		ProQOL – secondary traumatic stress	+0.10 ± 3.51, *p* > 0.05
**Mind–body interventions**			
Ibrahim (48)	Some concerns	Warwick-Edinburgh Mental Wellbeing Scale	INT: +2.4 ± 3.5; CON: +1.3 ± 6.9; *p* = NR
Keng (51)	Some concerns	ProQOL – compassion satisfaction (*T2*)	+0.053 (SD: NR), *p* = 0.483
		ProQOL – compassion satisfaction (*T3*)	**+0.183 (SD: NR), *p* = 0.007**
Liu (53)	Some concerns	WHOQoL-BREF – Physical health	**+10.1 (8.2, 12.0)**
		WHOQoL-BREF – Psychological	**+10.5 (8.1, 12.9)**
		WHOQoL-BREF – Social relationships	**+8.3 (6.7, 9.9)**
		WHOQoL-BREF – Environment	+0.9 (−1.1, 2.9)
Si (54)	High	Life Satisfaction Scale	**INT: +8.9 ± 2.7; CON: +0.1 ± 2.4; *p* < 0.001**
Yildirim (57)	Some concerns	Previously developed psychological wellbeing scale	**+5.15 (1.27, 9.03)**
**Workplace-based interventions**			
Zaghini (60)	Low	Nurses QoL	**+0.08 ± 0.3, *p* = 0.003**
**Resilience and coping**			
**Psychological support techniques**			
Jing (27)	Low	CD-RISC	+ 1.15 (−4.53, 2.23)
Klomp (29)	High	Self-efficacy to handle challenging situations	**+ 0.30 (0.24, 0.35)**
**Psychoeducation**			
Gnanapragasam (36)	Some concerns	BRS	+ 0.06 (−0.05, 0.16)
Leitch (38)	Some concerns	Coping (4-item, study-specific scale)	−0.12 (−0.47, 0.23)
		Resiliency (7-item, study-specific scale)	**+1.0 (0.6, 1.3)**
Powell (41)	Some concerns	Coping Self-Efficacy Scale	+4.7 ± 27.4 *p* > NR
Powell (42)	High	Coping strategies (single item, Likert scale from 1, not at all to 5, a lot)	**+0.70 ± 0.55, *p* < 0.01**
Yi-Frazier (44)	Low	CD-RISC	**+1.74 (1.00, 2.48)**
**Mind–body interventions**			
Joshi (50)	Some concerns	CD-RISC	+1.5 (−0.6, 3.7)
**Mood-related outcomes**			
**Psychological support techniques**			
Cole (24, 32, 33)	Low	5-item Dimensions of Anger Reaction (*T2*)	No significant difference
		5-item Dimensions of Anger Reaction (*T3*)	**−3.17 ± 3.80, *p* < 0.01**
**Mind–body interventions**			
Iwakuma (49)	High	Temporary Mood Scale – Anger	**Baseline, median: 15 (IQR: 12–15), End of study, median: 15 (IQR: 15–15), *p* = 0.018**
		Temporary Mood Scale – Confusion	**Baseline, median: 14 (IQR: 11–15), End of study, median: 15 (IQR: 15–15), *p* = 0.001**
		Temporary Mood Scale – Depression	**Baseline, median: 12 (IQR: 11–15), end of study, median: 15 (IQR: 15–15), *p* = 0.005**
		Temporary Mood Scale – Fatigue	**Baseline, median: 10 (IQR: 8–13), end of study, median: 15 (IQR: 13–15), *p* = 0.001**
		Temporary Mood Scale – Strain	**Baseline, median: 10 (IQR: 6–13), end of study, median: 14 (IQR: 13–15), *p* = 0.003**
		Temporary Mood Scale – Vigour	**Baseline, median: 5 (IQR: 4–9), end of study, median: 9 (IQR: 6–10), *p* = 0.01**
**Organizational**			
Saqib (59)	Some concerns	Self-reported mood (single item) before and after session	Sad: −37.62% Neutral: −53.12% Happy: +90.75% (*p* = NR)
**Other interventions**			
Giordano (61)	Some concerns	Self-reported mood (study-specific tool)	**Breathing, energy, and customized energy playlists decreased tiredness, sadness, fear, and worry (all *p* < 0.05, data NR)** **Customized breathing, customized energy playlists decreased sadness, fear, and worry (all *p* < 0.05, data NR);** no difference in tiredness

Brief Resilience Scale; CAPS, Clinician-Administered PTSD Scale for DSM-5; CD-RISC, Connor-Davidson Resilience Scale; CES-D, Center for Epidemiologic Studies Depression Scale; CI, Confidence interval; CON, Control group; DASS-21, Depression, Anxiety, Stress Scale; DSM, Diagnostic and Statistical Manual of Mental Disorders; GAD-7, Generalised anxiety disorder 7 scale; GHQ-12, General Health Questionnaire; HCP, Healthcare provider; INT, Intervention group; IQR, Interquartile range; MBI, Maslach Burnout Inventory; NR, Not reported; OR, Odds ratio; PCL-C, PTSD Checklist-Civilian version; PCL-S, PTSD Checklist-Specific Version; PHQ-9, Patient Health questionnaire 9; ProQOL, Professional Quality of Life; QoL, Quality of life; SLC-90, Symptom Checklist 90 scale; SMD, Standardized mean difference; STAI, State Trait Anxiety Inventory; PTSD, Post-traumatic stress disorder; SE, Standard error; SPRINT-E, Short Post-Traumatic Stress Disorder Rating Interview; WHOQoL-BREF, World Health Organization Quality of Life Brief Version.

**Bold** indicates a statistically significant difference.

### 3.3 Mental health-related outcomes by intervention type

#### 3.3.1 Psychotherapy

Nine studies were included that used psychotherapy to support healthcare workers’ mental health: three RCTs (25, 26, 34) and six quasi-experimental studies (24, 27–31). The components of the psychotherapy varied widely: five included CBT (24–26, 30, 34), two used psychological first aid (28, 29), one focused on mindfulness-based stress reduction (31), and one on acceptance and commitment therapy (27). Interventions were delivered in groups (*n* = 5, 56%) (24, 27, 29–31), one-on-one (*n* = 2, 22%) (26, 28), and electronically (*n* = 2, 22%) (25, 34). Interventions varied from a single session (28) to 12 weeks in duration.

**Figure 4:**
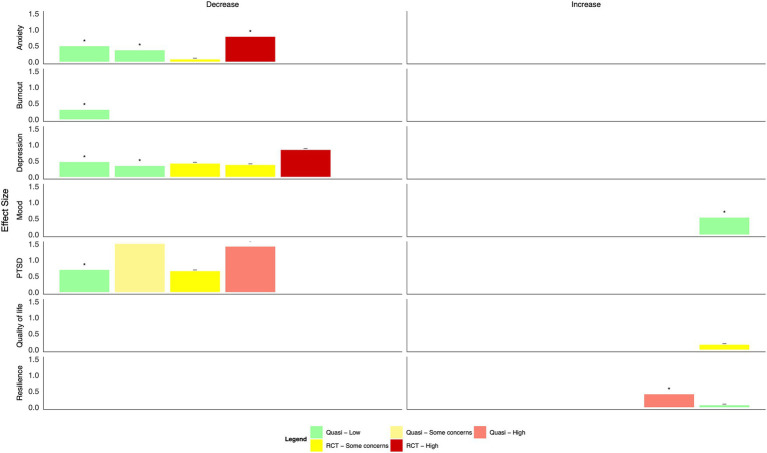
Harvest plot of the effects of psychotherapy on mental health outcomes.

Overall positive effects were reported across mental health outcomes (Figure 4, Table 2). However, most studies had some concerns or a high risk of bias. Many were not statistically significant: group CBT during Ebola and e-CBT during COVID-19 both significantly decreased anxiety scores with small–moderate effect sizes (24, 34), but a CBT- and positive-psychology-focused app during COVID-19 and group-based Acceptance and Commitment Therapy (ACT) did not (25, 27). Group-based CBT during Ebola also significantly reduced depression with small to moderate effect sizes, as did group-based ACT (24, 27); however, this was not seen in interventions of a CBT and positive psychology-based app or e-CBT during COVID-19 or in-person CBT in response to the September 11th attacks (25, 26, 34). Symptoms of PTSD generally improved across four studies with moderate to large effect sizes (24, 26, 28, 30); however, in one study that compared the effect of in-person group CBT following a natural disaster, those with and without diagnosed PTSD only improved in the group with a clinical diagnosis of PTSD (30). Only a single study each found statistically significant improvements in burnout (31), mood (24), and coping self-efficacy (29) with small effect sizes following mindfulness-based stress reduction, group-based CBT, and psychological first aid training, respectively; a single study also explored quality of life (25), with no statistically significant effects found using a CBT and positive psychology-based app.

#### 3.3.2 Psychoeducation

Ten studies were identified that used psychoeducation to promote healthcare workers’ mental health: four RCTs (35, 36, 39, 45) and six quasi-experimental studies (37, 38, 40–42, 44). All interventions included workshops or educational materials; 60% (*n* = 6) focused on physical stress management (35, 36, 38–40, 45). Few incorporated direct support from a mental health professional (*n* = 4, 40%) (35, 37, 38, 44), peer support (*n* = 2, 20%) (37, 42), or a basis in CBT (*n* = 2, 20%) (35, 36). Half of the interventions consisted of only one session (37, 39–42), with the longest lasting 12 weeks (45). While two interventions (20%) were self-paced apps (35, 36), most were delivered face-to-face (*n* = 6, 60%) (37–39, 41, 42, 45) and in a group setting (*n* = 7, 70%) (37–39, 41, 42, 44, 45).

**Figure 5:**
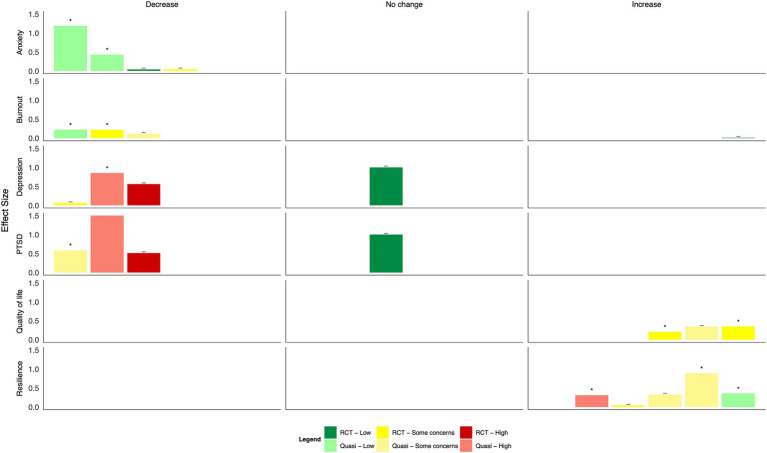
Harvest plot of the effects of psychoeducation on mental health outcomes.

Generally, psychoeducational interventions promoted positive effects on mental health outcomes, although many changes were not statistically significant (Figure 5, Table 2). Both a virtual reality-based stress management intervention and resilience coaching program studies with low risk of bias found moderate to large statistically significant reductions in anxiety (40, 44); two app-based psychoeducation interventions found small, non-significant decreases (35, 36). Four studies explored burnout. Two group-based psychoeducation interventions resulted in small, statistically significant decreases (44, 45). In contrast, a single, group-based session did not result in a statistically significant change. HCWs who used a CBT-based app reported slightly higher burnout scores at the end of the intervention (35). Moderate-to-large reductions in depression and PTSD symptoms were observed after using a psychoeducation app and group-based workshops (36, 37, 39). However, differences were generally not statistically significant; a fourth study of an app-based intervention found no change in either outcome (35). Concerning positive mental health outcomes, group-based resilience workshops and a mental wellbeing app resulted in moderate improvements in quality of life, although with some concerns of risk of bias (36, 45). A similar improvement was found following a resilience-focused group-based workshop, although the findings were not statistically significant (41). Resilience measures improved following group-based resilience training (38, 44) and group-based stress reduction (42) with moderate to large effect sizes. A single resilience training workshop and app-based intervention did not result in statistically significant improvements in resilience among HCWs (36, 41).

#### 3.3.3 Mind–body interventions

Twelve mind–body interventions were identified: seven RCTs (46, 47, 50–52, 54, 57) and five quasi-experimental studies (48, 49, 53, 55, 56). Nearly all interventions included a focus on physical stress management (*n* = 10, 91%) (46, 48–57), and over half of the interventions had an educational workshop (*n* = 6, 55%) (46, 49, 50, 53, 55, 56) or educational materials (*n* = 6, 55%) (46, 48, 51, 54–56). Only two (18%) involved interaction with a mental health professional (46, 50), and none incorporated peer support. The length of interventions ranged from a single session (49, 57) to 8 weeks (55, 56). Interventions were predominantly delivered in groups (*n* = 8, 73%) (48–50, 52–57), with one (9%) (51) provided asynchronously via a mobile application and two (18%) (46, 47) not reporting whether interventions were delivered in group or individual format. Five interventions (45%) used virtual delivery (46, 48, 51, 52, 54), and five (45%) were delivered in person (47, 49, 53, 55, 56); one did not report the mode of delivery (50).

**Figure 6:**
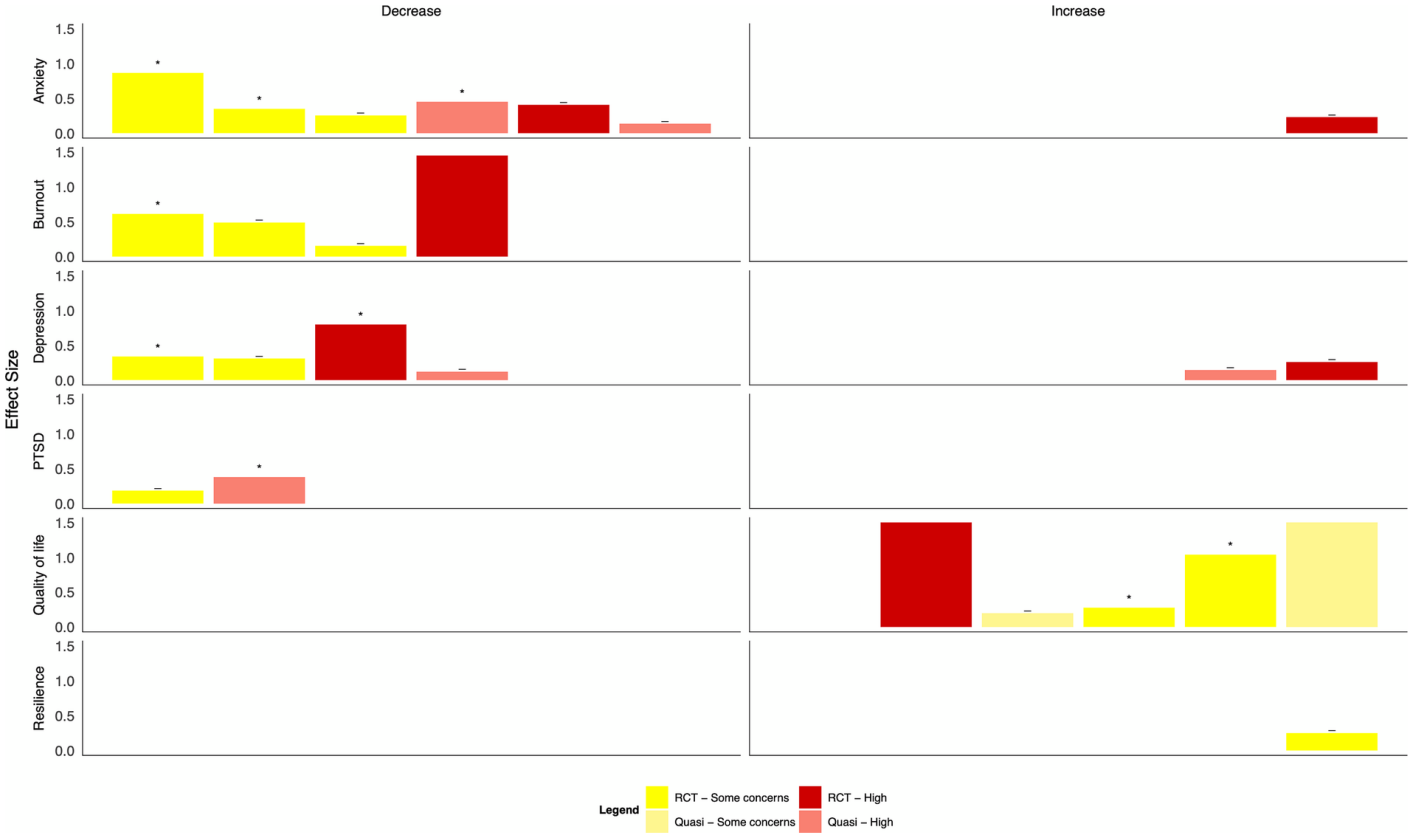
Harvest plot of the effects of mind–body interventions on mental health outcomes.

Findings from mind–body interventions suggest moderate to large reductions in anxiety, burnout, and depression, and small to moderate decreases in PTSD as well as large increases in quality of life; however, many did not reach statistical significance, and all studies had some concerns or a high risk of bias (Figure 6, Table 2). Of eight studies that measured anxiety, statistically significant improvements were found following a transcendental meditation intervention, a meditation retreat followed by a home study program following Hurricane Katrina, and a mindfulness-based breathing and music therapy intervention (50, 56, 57). These improvements did not reach statistical significance in interventions consisting of mindfulness training for social workers (46), a mindfulness app (51), a Ba Duan Jin intervention (53), or a meditation retreat and home practice following Typhoon Haiyan (55). In an RCT of brief mindfulness meditation, decreases in anxiety following the intervention period were large in the control group (52). Significant reductions in burnout were reported following Gong meditation (47), transcendental meditation (50), and laughter yoga (54), but not following a meditation app (51).

Statistically significant decreases in depression were only found following mindfulness training plus daily meditation (46); moderate effect sizes were found following transcendental meditation (50), a meditation app (51), and meditation retreats following Hurricane Katrina (56). In one RCT of brief mindfulness meditation and a quasi-experimental study of a meditation retreat, depression scores were higher in the intervention vs. comparator group (52, 55). Concerning PTSD symptoms, a meditation retreat following home practices resulted in statistically significant reductions (56); however, reductions were not significant following the use of a mindfulness app (51). Ba Duan Jin exercises (53), laughter yoga (54), and mindfulness breathing and music therapy (57) resulted in large and statistically significant improvements in quality of life; these improvements were not significant following a mindfulness breathing intervention delivered via WhatsApp (48) or the use of a meditation app (51). Only one study explored the impact of transcendental meditation on resilience, which did not find a statistically significant difference between groups (50).

#### 3.3.4 Organizational interventions

**Figure 7:**
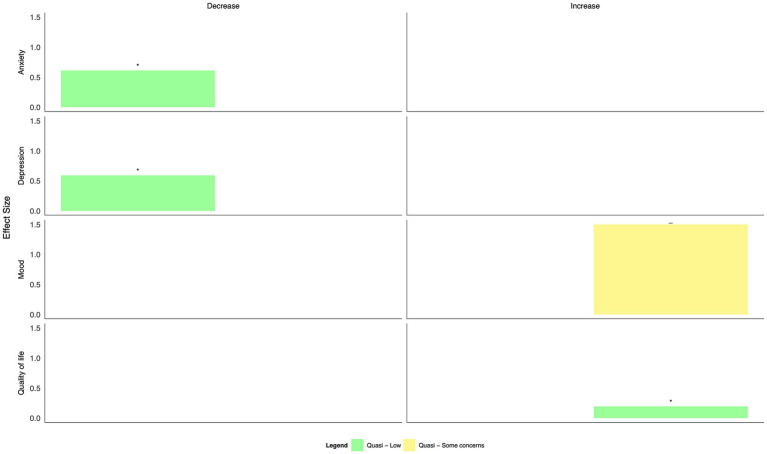
Harvest plot of the effects of organizational interventions on mental health outcomes.

Three different interventions were delivered at the organizational level during the 2003 SARS pandemic in Taiwan (58) and the COVID-19 pandemic in the UK (59) and Italy (60), all with low or some concerns concerning the risk of bias (Figure 7, Table 2). These interventions included enhanced training and surveillance infection prevention and control measures (58, 60), reorganization of care and staffing levels (58, 60), and supports such as yoga, mindfulness, and general mental health support (58, 59). The effect of these changes was evaluated after 1 month (59) and up to 5 months (60). An intervention focused on staffing, infection prevention and control measures, and mental health supports resulted in statistically significant reductions in anxiety and depression; effect sizes were moderate, and the risk of bias was low (58). An intervention delivering a mental health support hub within the hospital resulted in a large improvement in mood but was not statistically significant, and there were some concerns about the risk of bias (59). Finally, an intervention including proactive management, reorganized care settings, an increase in staffing, and online education resulted in small but statistically significant improvements in quality of life among nurses (60).

#### 3.3.5 Other intervention types

Finally, two interventions were identified that did not fall into one of the categories above. Giordano et al. used trained music therapists to develop relaxation playlists and listening guides to reduce stress and anxiety and improve energy and concentration among HCWs in Italy during the COVID-19 pandemic, with some concerns about the risk of bias (61). After 5 weeks, playlists decreased tiredness, sadness, fear, and worry compared to pre-intervention. Mahdood et al. tested aromatherapy before a work shift and during sleep on HCW with anxiety and insomnia during the COVID-19 pandemic in Iran; some concerns were identified with the risk of bias (62). Those in the intervention group displayed statistically significant reductions in anxiety compared to the control group after 30 days.

### 3.4 Mental health outcomes by delivery mode

**Figure 8:**
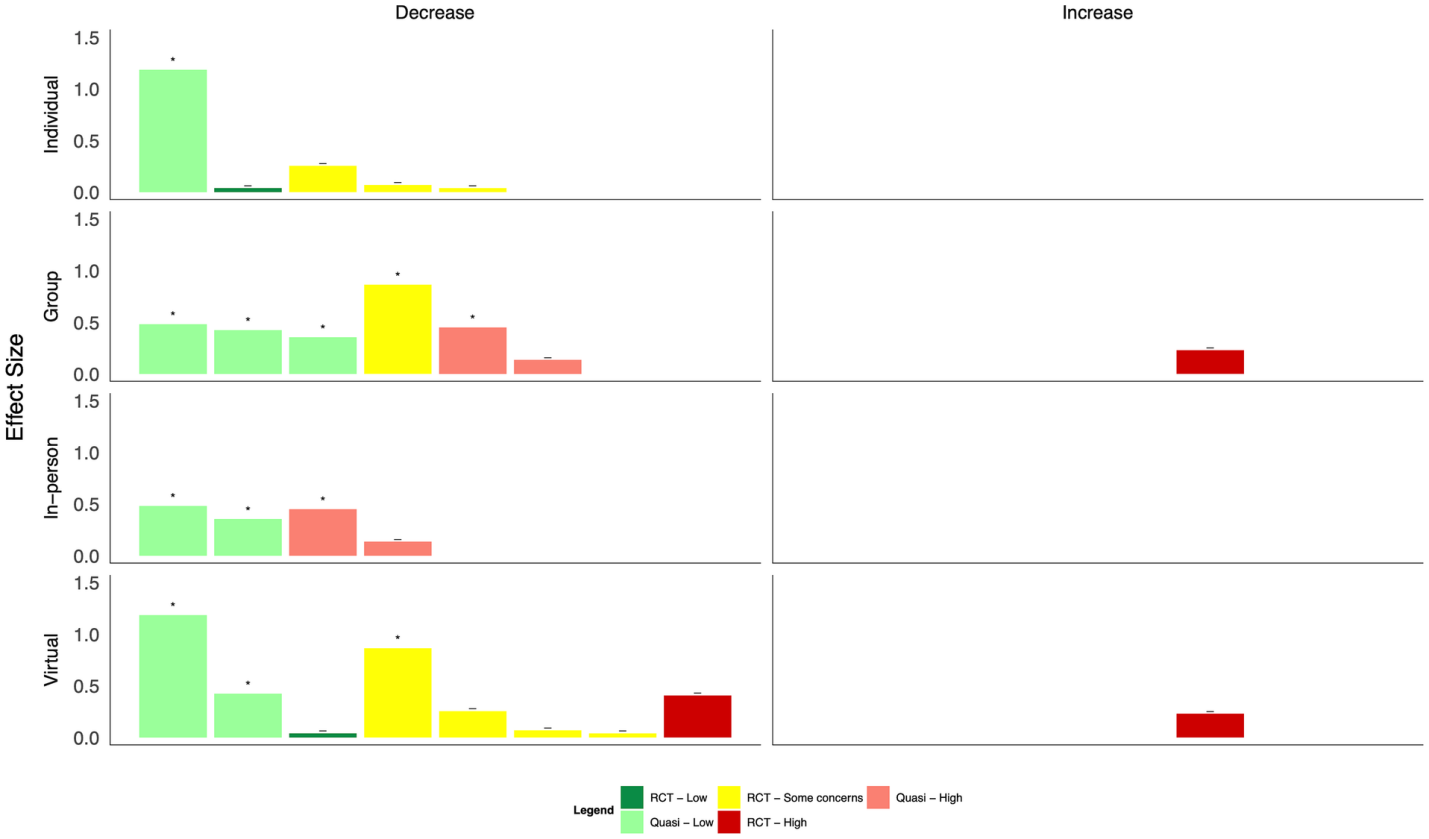
Harvest plot of changes in anxiety by mode of delivery.

**Figure 9:**
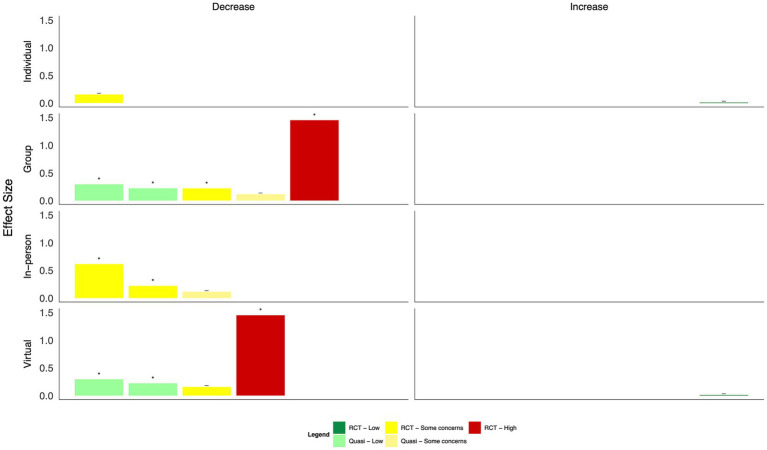
Harvest plot of changes in burnout by mode of delivery.

**Figure 10:**
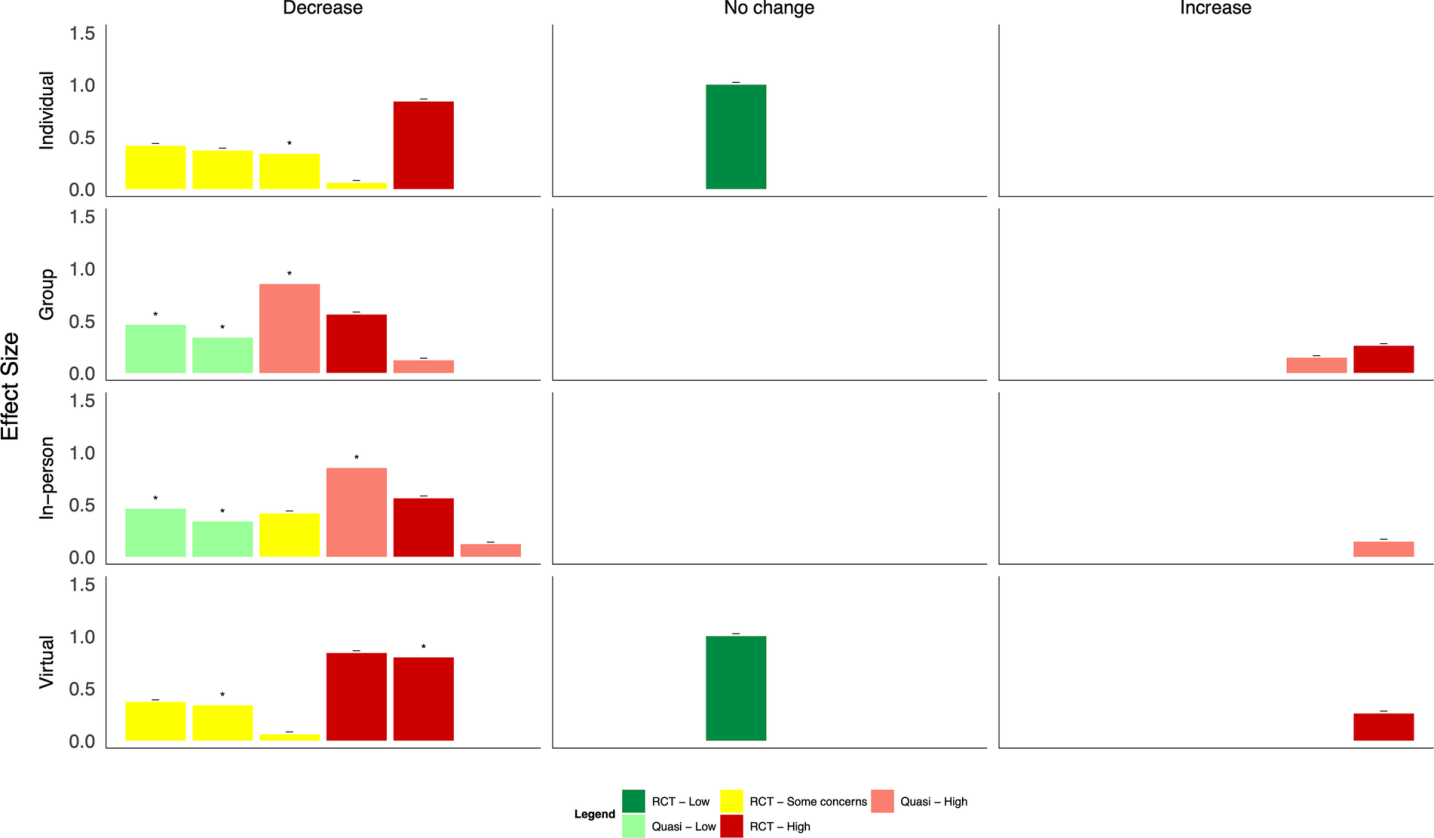
Harvest plot of changes in depression by mode of delivery.

**Figure 11:**
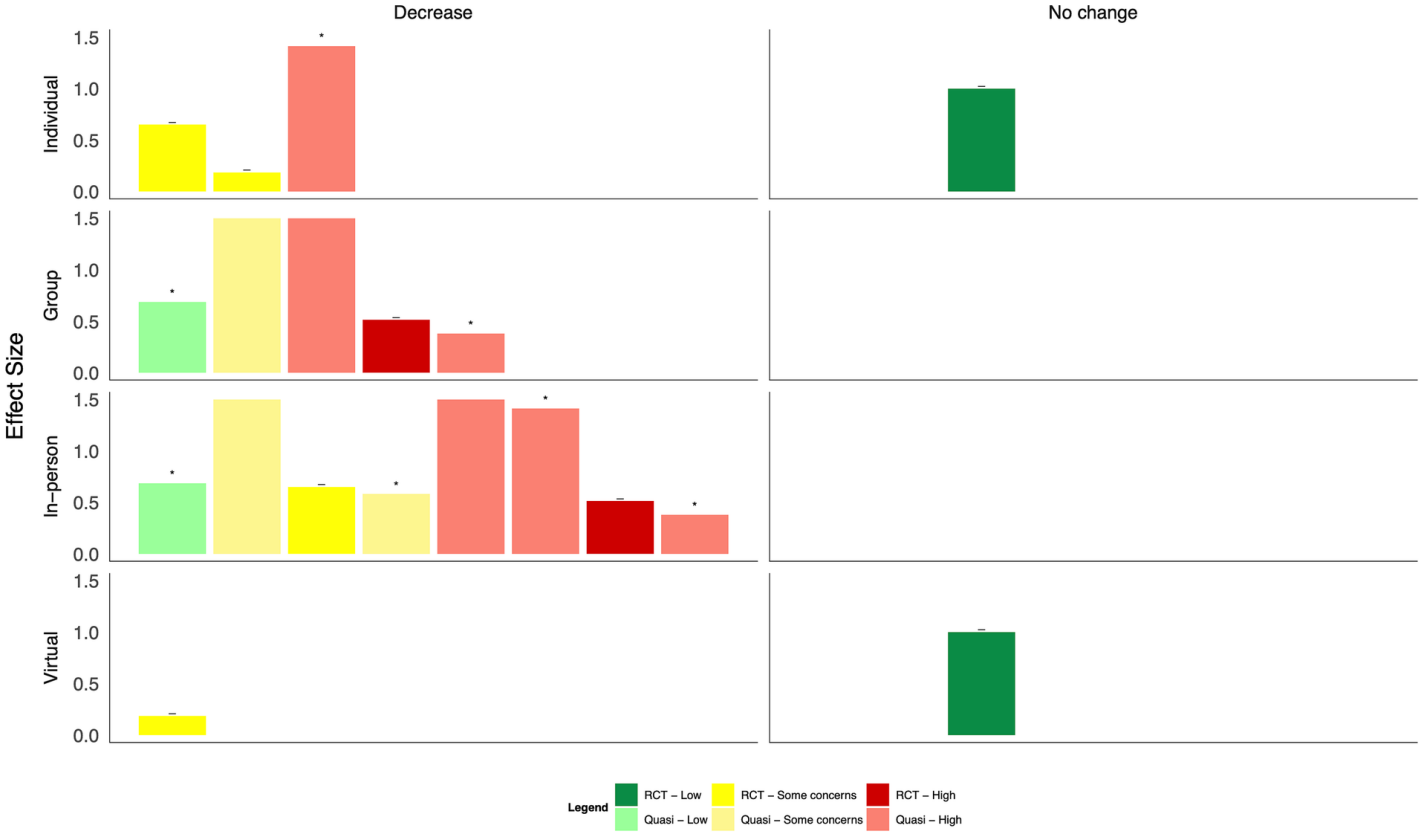
Harvest plot of changes in Post traumatic stress disorder by mode of delivery.

**Figure 12:**
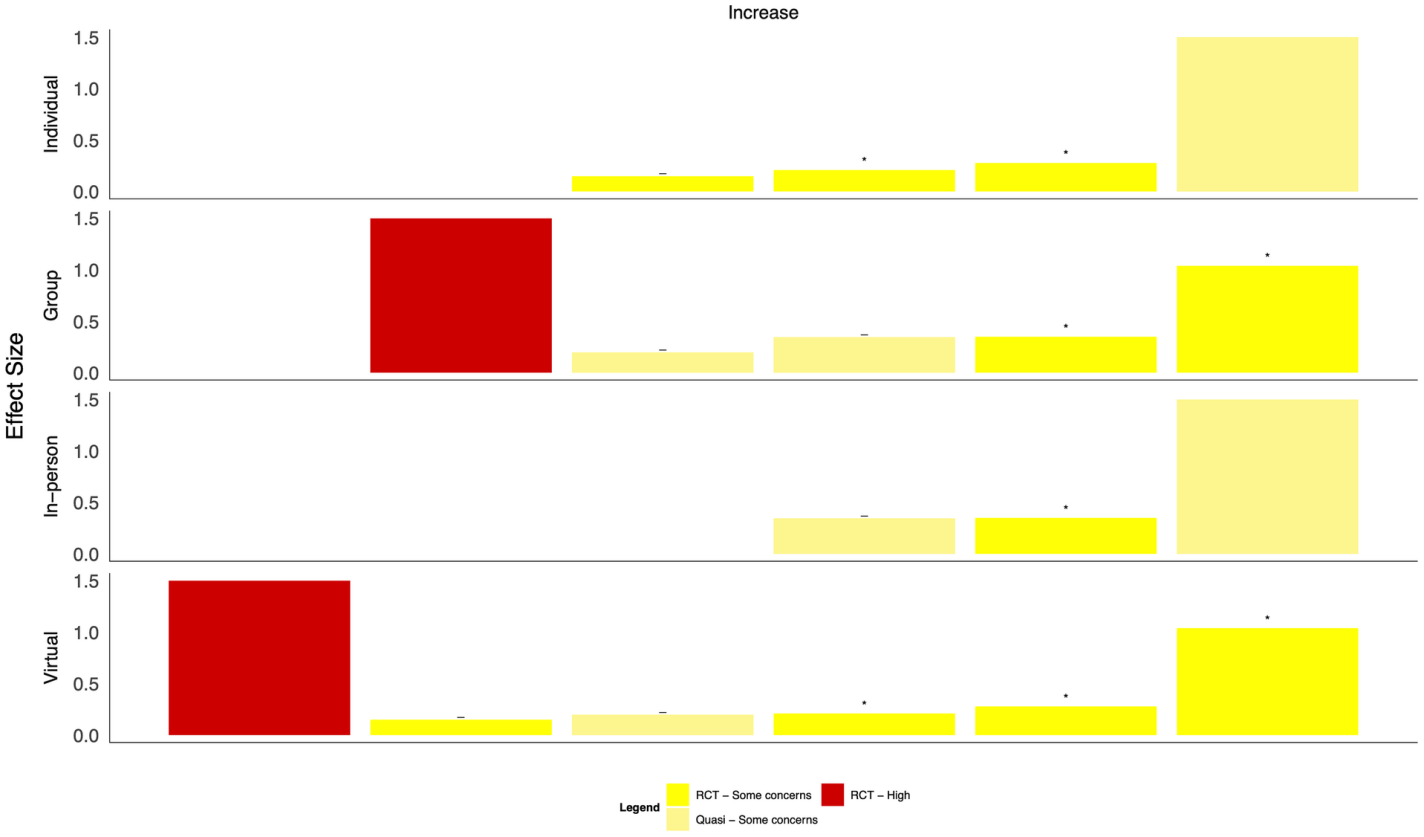
Harvest plot of changes in quality of life by mode of delivery.

**Figure 13:**
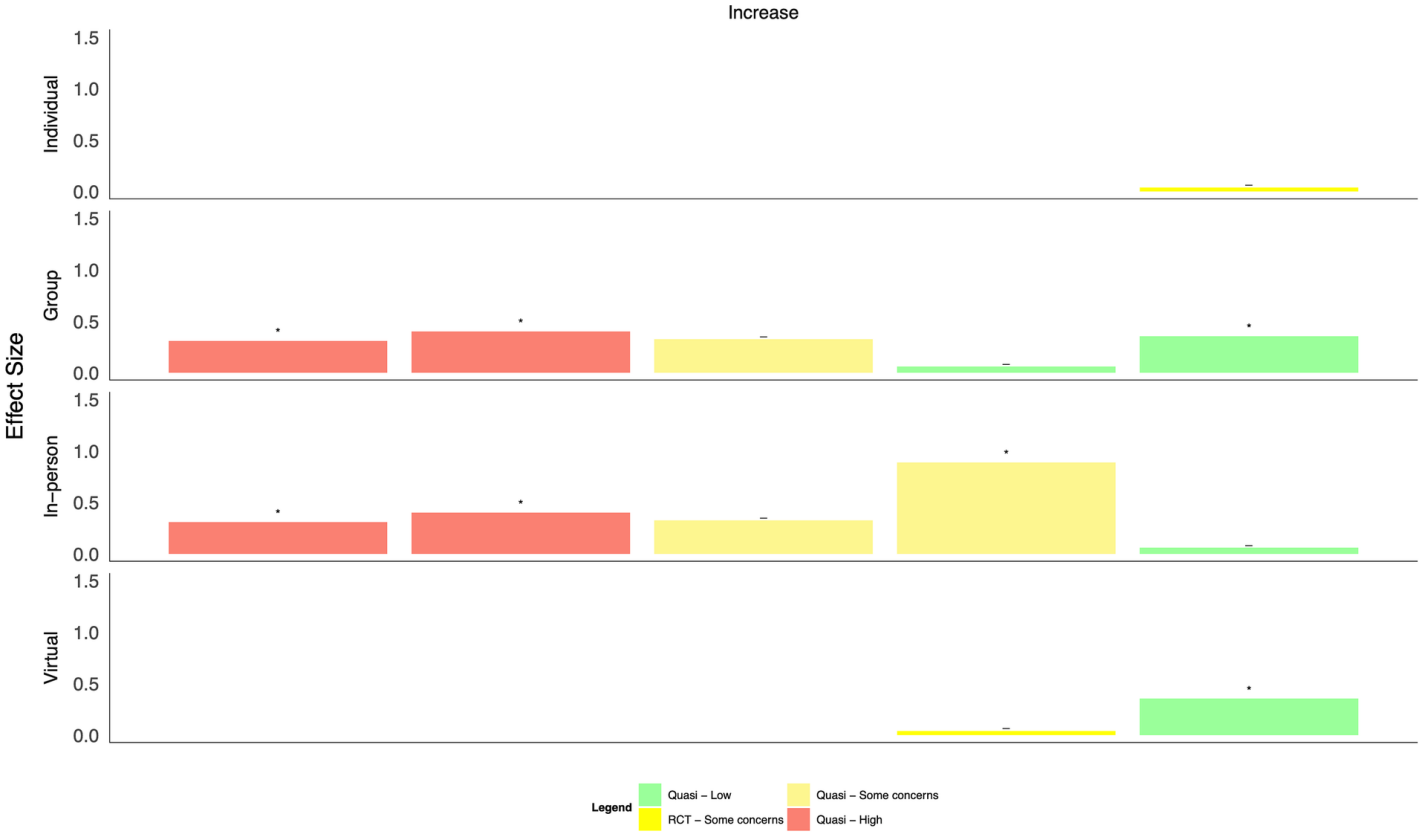
Harvest plot of changes in resilience by mode of delivery.

When intervention categories were combined, few clear patterns emerged for differences in effect size by mode of delivery (in-person, virtual, individual, and group-based, Figures 8–13). With respect to anxiety, interventions that were delivered in a group-based format were more consistently statistically significant than interventions delivered individually, with effect sizes generally moderate, and in-person compared to virtually delivered interventions had more consistently positive findings for reductions in depression. In the remaining comparisons, there were either similar findings by mode of delivery or too few studies in one or more categories to make meaningful comparisons.

## 4 Discussion

This review examined the use and effectiveness of strategies implemented at the individual and organizational levels to mitigate the psychological impacts experienced by those responding to a public health emergency. Recent systematic reviews focused on the COVID-19 pandemic have reported on the growing prevalence of adverse mental health outcomes in HCWs, which adds to the body of literature from previous public health emergencies (63–68). In addition to documenting the burden, there is also a need to identify effective interventions that can be used in recovery from the early, acute phases of the pandemic and a knowledge of the types of interventions that could be efficiently implemented in future public health emergencies.

This review found that psychotherapy had the strongest effect on decreasing several negative mental health outcomes and that psychoeducation may also be effective. Mind–body interventions seem particularly promising for improving quality of life, but more high-quality research is needed. These findings are consistent with the findings of a rapid synthesis of strategies to support the mental health and resilience of the public health workforce (including studies that were not focused on public health emergencies) (69). This review found that effective interventions were multi-sessional, built on existing evidence-based practices such as CBT, aimed to increase knowledge and skills in safety, problem-solving, resilience, and coping, and incorporated aspects of physical stress management. Another review focused on only eHealth interventions to reduce stress and promote the mental health of healthcare professionals and also found preliminary effectiveness for ‘third wave’ psychotherapies, such as mindfulness, compassion, and acceptance therapies. Like in this review, high heterogeneity was found across studies (70). Finally, a 2020 Cochrane review found very low certainty evidence that resilience training, which included mindfulness and cognitive behavioral therapy, may result in higher resilience and lower depression and stress in healthcare professionals. Importantly, this review only included studies published up to June 2019, thus did not include important lessons learned during the COVID-19 pandemic.

A future focus on widespread organizational-level interventions is also important to inform future emergency response. While individual-level interventions do appear to have positive impacts, there may be important equity and access implications. Many of the interventions tested during the COVID-19 pandemic were offered virtually, given widespread public health protocols in place to reduce the spread of infection; this mode of delivery may have also enhanced the participation of HCWs who may be unable or unwilling to take part in in-person programming. An important factor that is not considered in research-tested interventions is the cost of delivery. Out-of-pocket expenses for mental health treatment by a qualified professional may be prohibitive for many HCWs. Similarly, there is a need for more cultural competency in psychological interventions (i.e., CBT and PFA). Differing cultural perspectives may lead to conflicting views on how some think about and understand mental health and wellness, and research literature has highlighted the importance of considering cultural components in the spread and scale of evidence-based mental health interventions (71). Recognizing diversity within the workforce is a critical step in providing adequate mental health support.

Based on the visual inspection of the harvest plots, the data suggest that group-based interventions may be more effective than individually delivered interventions, including one-on-one intervention delivery or delivery via the internet or mobile applications, suggesting that a sense of group cohesion is relevant to effective interventions. This is in line with data from qualitative reviews, which have identified feelings of camaraderie and team building as important components that may mediate the efficacy of the intervention on individuals’ mental health (72, 73). A recently published scoping review synthesized evidence on the key concerns of healthcare providers with respect to COVID-19 (74). Based on the findings, the authors recommended timely and personalized mental health support, particularly through interdisciplinary teams, to minimize burnout; this recommendation is supported by the findings presented here.

To answer our research question, we employed a comprehensive search strategy of multiple databases and gray literature and followed best practices for evidence synthesis, following the Cochrane Handbook for Systematic Review of Interventions to reduce bias in our study. The certainty of the findings of this review is limited by the quality of the included single studies. Most studies were quasi-experimental, using a pre-test and post-test design rather than RCTs. This is not surprising given that many were evaluations of real-world programs that were implemented in response to an unexpected public health emergency and were not pre-designed for the purpose of determining intervention efficacy. Thus, positive findings from studies without an appropriate control group may be confounded by other factors. At the same time, these real-world evaluations may be more generalizable than studies that are conducted in tightly controlled trial conditions. Within the included studies, effect sizes do appear to be larger in studies with a higher risk of bias. A large loss to follow-up was observed across both RCTs and quasi-experimental studies, which may overinflate the effect estimates if those who withdraw from a study are those who are not experiencing a benefit of the intervention. Heterogeneity within categories was high, precluding the use of statistical techniques such as meta-analyses to estimate effect sizes more precisely. These factors should be considered when using the evidence presented here in decision-making.

In addition, studies measuring anxiety, burnout, depression, and PTSD included in this review rarely provided a diagnostic criterion for inclusion. It is possible that the types of interventions needed to effectively treat a clinical diagnosis of a mental health disorder such as generalized anxiety disorder or major depressive disorder may be different than the type of intervention needed to reduce the risk of developing such disorders. Further studies should clearly describe recruitment strategies and eligibility criteria and perform subgroup analyses by baseline scores to better understand whether tailoring of interventions may be needed based on one’s baseline mental health status. This is particularly important for including diverse populations who are less likely to acknowledge psychological symptoms and report being concerned about the stigma associated with mental illness (75). Finally, most of the interventions included were of relatively short duration; the medium- or long-term impact of these interventions is unknown.

## 5 Conclusion

This review adds to a growing body of evidence on strategies to mitigate the adverse psychological effects of frontline HCWs responding to a variety of public health emergencies, including pandemics and natural disasters. This review highlights the need for ongoing, rigorous research and evaluation of mental health programming implemented at the individual and organizational levels to mitigate the mental health risks in future pandemics or public health emergencies. Strategies to improve the mental health and wellbeing of HCWs following the COVID-19 pandemic will not be a “one size fits all” approach; however, the data to date elucidate promising areas to invest in to support the mental health of HCWs.

## Data availability statement

The original contributions presented in the study are included in the article/Supplementary material, further inquiries can be directed to the corresponding author.

## Author contributions

SN-S: Conceptualization, Data curation, Formal analysis, Investigation, Methodology, Project administration, Supervision, Validation, Visualization, Writing – original draft, Writing – review & editing. EB: Conceptualization, Data curation, Methodology, Supervision, Writing – review & editing. SH: Conceptualization, Formal analysis, Project administration, Writing – original draft, Writing – review & editing. DS: Methodology, Writing – review & editing. LA: Methodology, Writing – review & editing. EA: Methodology, Writing – review & editing. LK: Methodology, Writing – review & editing. JT: Methodology, Writing – review & editing. OB: Methodology, Writing – review & editing. SK: Methodology, Writing – review & editing. SM: Methodology, Writing – review & editing. MD: Conceptualization, Funding acquisition, Investigation, Methodology, Supervision, Validation, Writing – original draft, Writing – review & editing.
