# Supplementary material for: What are effective strategies to respond to the psychological impacts of working on the frontlines of a public health emergency?
Source: Front Public Health. 2023 Nov 7;11:1282296. doi: 10.3389/fpubh.2023.1282296 (PMC10733471; doi:10.3389/fpubh.2023.1282296)
Supplement: Supplementary file 1 [file Data_Sheet_1.docx]

**Supplemental Figure 1: Medline search strategy**

| 1. | health care personnel/ |
| --- | --- |
| 2. | "health personnel".mp. |
| 3. | "allied health professional*".mp. |
| 4. | ("infection control professional*" or "infection control practition*").mp. |
| 5. | (nurs* or nurse practitioner*).mp. |
| 6. | (physician* or doctor*).mp. |
| 7. | (health care worker* or healthcare worker*).mp. |
| 8. | (personal support worker* or PSW).mp. |
| 9. | (occupational therapist* or physiotherapist* or social work* or physical therapist*).mp. |
| 10. | public health service/ |
| 11. | public health*.mp. |
| 12. | community health nursing/ |
| 13. | nursing home/ |
| 14. | residential home/ |
| 15. | home care/ |
| 16. | (long term care* or long-term care* or longterm care* or LTC or nursing home* or long term facilit* or residential facilit* or assisted living* or group home* or home for the age* or homes for the age*).mp. |
| 17. | (home care* or community care*).mp. |
| 18. | public health/ |
| 19. | preventive medicine/ |
| 20. | community health*.mp. |
| 21. | health care organization/ |
| 22. | health maintenance organization/ |
| 23. | hospital organization/ |
| 24. | organization/ |
| 25. | "organization and management"/ |
| 26. | ((health* adj2 organization*) or (health* adj2 system*)).mp. [mp=title, abstract, heading word, drug trade name, original title, device manufacturer, drug manufacturer, device trade name, keyword, floating subheading word, candidate term word] |
| 27. | ((hospital* adj2 organization*) or (hospital* adj2 system*)).mp. |
| 28. | or/1-27 |
| 29. | epidemic/ |
| 30. | pandemic/ |
| 31. | influenza/ or pandemic influenza/ or swine influenza/ |
| 32. | spatiotemporal analysis/ |
| 33. | (disease outbreak* or infection outbreak* or epidemic* or pandemic*).mp. |
| 34. | (H1N1 or swine flu or SARS or severe acute respiratory syndrome or MERS or middle eastern respiratory syndrome or influenza*).mp. |
| 35. | disaster medicine/ or disaster/ or disaster planning/ or natural disaster/ |
| 36. | emergency/ |
| 37. | medical countermeasure/ |
| 38. | (disaster* or public health cris* or public health emergenc* or natural disaster*).mp. |
| 39. | (covid* or corona*).mp. |
| 40. | or/29-39 |
| 41. | occupational health/ or occupational health service/ |
| 42. | (occupational health* or employee health* or staff health* or employee well being* or employee wellbeing* or staff well being* or staff wellbeing* or employee assistan* or staff assistan*).mp. |
| 43. | counseling/ |
| 44. | meditation/ |
| 45. | relaxation training/ |
| 46. | imagery/ |
| 47. | guided imagery/ |
| 48. | laughter therapy/ |
| 49. | (meditat* or mental healing* or relaxation therap* or imagery or laughter therap*).mp. |
| 50. | mental health service/ |
| 51. | (mental health service* or mental health support* or psychological first aid or psychoeducation or psychosocial or debreif* or protective factor*).mp. |
| 52. | mindfulness/ |
| 53. | mindfulness*.mp. |
| 54. | cognitive behavioral therapy/ |
| 55. | (cognitive behavioural therap* or cognitive behavioral therap*).mp. |
| 56. | "eye movement desensitization and reprocessing"/ |
| 57. | "desensitization (psychology)"/ |
| 58. | (Eye Movement Desensitization Reprocessing or EMDR or desensitiz* or trauma treat* or trauma intervention* or trauma therap* or crisis reponse*).mp. |
| 59. | (counsel* or talk therap*).mp. |
| 60. | (social support* or self care* or self-care* or breathing technique* or calming technique* or distraction*).mp. |
| 61. | ((organization* or institution* or workplace* or system*) adj3 (intervention* or strateg* or program* or prevent* or support*)).mp. |
| 62. | or/41-61 |
| 63. | coping behavior/ |
| 64. | (psychological adapt* or psychological heal* or resilien* or coping* or cope).mp. |
| 65. | mental health/ |
| 66. | (anxiety* or anxiety disorder* or bipolar* or bipolar disorder* or dissociat* or dissociative disorder* or mood disorder* or personality disorder* or psychotic disorder* or sleep wake disorder* or isomnia* or trauma* or stress*).mp. |
| 67. | (mental health* or mental disorder* or mental illness or psychiatric illness* or psychiatric disorder*).mp. |
| 68. | health personnel attitude/ or attitude/ or attitude to health/ |
| 69. | catastrophizing/ |
| 70. | optimism/ |
| 71. | pessimism/ |
| 72. | (optimis* or pessimis* or catastrophiz*).mp. |
| 73. | stress/ or acute stress/ or adaptation syndrome/ or behavioral stress/ or burnout/ or caregiver burden/ or chronic stress/ or critical incident stress/ or emotional stress/ or family stress/ or home stress/ or interpersonal stress/ or job stress/ or life stress/ or mental stress/ or role stress/ |
| 74. | (burnout* or occupational stress* or compassion fatigue* or mental fatigue or fatigue*).mp. |
| 75. | fatigue/ or exhaustion/ |
| 76. | dysthymia/ |
| 77. | "alert fatigue (health care)"/ |
| 78. | compassion fatigue/ |
| 79. | emotional disorder/ |
| 80. | depersonalization/ |
| 81. | depression/ |
| 82. | (affective symptom* or affective disorder* or depersonaliz* or depression* or emotional impact*).mp. |
| 83. | (anger or angry or apathy or bereav* or boredom or emotional regulat* or psychological distress* or psychological impact* or fear* or frustrat* or guilt* or happy* or happiness or hope* or lonely or loneliness or sad* or greif* or griev* or mourn* or uncertain* or loss of control* or lack of control*).mp. |
| 84. | (post-traumatic stress disorder* or PTSD or psychological trauma or vicarious trauma* or moral injur* or moral disress* or panic* or absentee* or interpersonal conflict* or domestic violen* or acute stress disorder*).mp. |
| 85. | drug dependence/ |
| 86. | (substance abuse* or substance use* or alcohol use* or alcohol abuse* or drug use* or drug abuse*).mp. |
| 87. | sleep disorder/ |
| 88. | insomnia/ |
| 89. | (sleep or sleep disorder*).mp. |
| 90. | exp suicidal behavior/ |
| 91. | (suicid* or attempted suicide or self harm or violen*).mp. |
| 92. | emotion/ or affect/ or anger/ or boredom/ or emotion regulation/ or fear/ or frustration/ or grief/ or guilt/ or happiness/ or hope/ or unhappiness/ |
| 93. | irritability/ |
| 94. | apathy/ |
| 95. | distress syndrome/ |
| 96. | panic/ |
| 97. | loneliness/ |
| 98. | sadness/ |
| 99. | mental disease/ or addiction/ or anxiety disorder/ or dissociative disorder/ or mood disorder/ or personality disorder/ or schizophrenia spectrum disorder/ |
| 100. | depression/ or bipolar disorder/ or dysthymia/ or major depression/ |
| 101. | paranoid psychosis/ |
| 102. | posttraumatic stress disorder/ |
| 103. | schizophreni*.mp. |
| 104. | or/63-103 |
| 105. | 28 and 40 and 62 and 104 |

**Supplemental table 1. Modified TIDieR table**

| Name | Why | What \| materials and procedures | Workshop | Educational materials | Peer Support | MH Professional | PFA or debriefing | CBT | Physical stress management | Who provided | How | Virtual | Face-to-face | Individual | Group | When and how much | Fidelity  Planned \| Actual |
| --- | --- | --- | --- | --- | --- | --- | --- | --- | --- | --- | --- | --- | --- | --- | --- | --- | --- |
| Psychological support techniques (n = 9) | | | | | | | | | | | | | | | | | |
| Cole, 2021^1-3^  3-phased approach for HCW during Ebola | Although HCW are often resilient, there is evidence that working in disaster healthcare settings can elicit psychological difficulties including anxiety, depression, and PTSD. | 3-phased approach based on ongoing need  Phase 1: PFA workshop  Phase 2: ongoing workshops focused on ongoing mental health difficulties, psychoeducation, coping strategies based on behavioural and cognitive approaches  Phase 3: small group CBT | x | x | x | x | x | x | - | UK-based CBT facilitators (psychologist or psychotherapist) trained local peer-staff. | Large and small group workshops | x | x | - | x | Phase 1: single, 2h session  Phase 2: 0-6 sessions, 2h/session over 10 wks  Phase 3: 6 weekly sessions | NR |
| De Kock, 2022^4^  CBT and positive psychology-based app | Several barriers to seeking mental health treatment exist, especially for health care staff working through the pandemic. | Knowledge users informed adaptation of existing digital psychology app to be relevant to the COVID-19 pandemic. | - | x | - | - | - | x | - | NHS | Smartphone app | x | - | x | - | Self-directed use for 1 month | Average >1 use per day |
| Difede, 2007^5^  CBT for disaster workers following 9/11 | Multiple studies have documented rates of PTSD in disaster workers worldwide, ranging from 9% to 37%. | Previously developed CBT program, including:  (a) psychoeducation; (b) treatment rationale and contracting; (c) breathing exercises; (d) imaginal exposure; (e) gradual in vivo exposure; (f) cognitive reprocessing; (g) relapse prevention; (h) homework | x | x | - | x | - | x | x | Licensed psychologists and social workers with a minimum of 3 years of clinical experience who had received specific training. | CBT therapy sessions | - | x | x | - | 12 weekly 75-min session | Random sessions assessed for fidelity; no protocol violations noted |
| Jing, 2022^6^  Group-based ACT | National guidelines on emergency psychological crisis intervention for COVID-19 health care workers | Adaptation to previously developed Acceptance and Commitment Therapy + psychoeducation intervention for nurses | - | x | x | x | - | - | - | Mental health professor, neuropsychology physician, psychotherapist, psychological counsellor | Group-based sessions in staff rooms | - | x | - | x | 10 weekly 20-min sessions | Checklist was used to ensure fidelity (results NR) |
| Ke, 2017^7^  Debriefing and PFA post-earthquake | PTSD is common in disaster HCW; disaster related ASD and depression may cause functional impairment in HCW, which in turn affects the quality of care. | PFA, debriefing, minilectures to improve awareness of mental health; referral to psychiatrist as needed. Muscle and mental relaxation for awareness of physical stress. | x | - | - | x | x | - | x | Psychotherapists, psychiatrists, physical therapist | Session held at disaster site | - | x | x | - | Post-earthquake; single session at the end of each 8-h shift | NR |
| Klomp, 2020^8^  Resilience training for Ebola responders | Being concerned about the professionals aiding in the Ebola response, the CDC sought to strengthen the health, safety, and resilience of team members before, during and after deployment. | Pre and post deployment briefings, PFA, resilience training and a virtual reality environment exercise that simulated various types of emergencies. | x | - | x | - | x | - | x | Psychiatrists, resilience team | Large group-based sessions  VRE; small group session | x | x | - | x | 3-day course pre-deployment; 50 min VRE session | NR |
| Leiva-Bianchi, 2018^9^  CBT for post-disaster stress post-earthquake | Studies performed after the Chilean earthquake showed that HCW’s MH was negatively affected. | CBT-post-disaster is a short-term group therapy whose objective is to identify and to intervene in the maladaptive beliefs related to the disaster. CBT-PD included psychoeducation, breathing retraining, behavioural activation, and cognitive restructuring. Clients receive a workbook to complete assignments to reinforce the skills that they have learned in session. | x | x | - | x | - | x | - | 2 therapists with a degree in psychology and trained in the procedure | Group-based sessions | - | x | - | x | 10-12, 60-90 min sessions over 6 months | NR |
| Osman, 2021^10^  Brief, mindfulness-based intervention | HCP experiencing high emotional toll and burnout from COVID-19; full mindfulness-based stress reduction may be too intensive to complete | A brief version of mindfulness-based stress reduction delivered based on previously developed workbook. | - | - | - | x | - | - | x | Clinical psychologist and mindfulness teacher | Virtual, group-based sessions | x | - | - | x | 4, 1-hour sessions | NR |
| Zhou, 2022^11^  e-CBT for insomnia | Physiological and psychological stress due to the COVID-19 pandemic has impact sleep and mood of nurses. | Using SUBIAN CBT-I management system, trained, experienced staff created custom treatment and follow-up plans based on individual compliance and improvement. | - | - | - | x | - | x | x | Trained and experienced medical staff | Online platform | x | - | x | - | 1x/week, 6 weeks | NR |
| Psychoeducation interventions (n = 10) | | | | | | | | | | | | | | | | | |
| Berger, 2011^12^  Weekly psychoeducation sessions during Lebanon war | Nurses who treat victims of conflict are more vulnerable to professional burnout and are at risk for developing secondary traumatization. | - Experiential exercises, homework assignments, educational resources. - Workshops included information about attachment theory, development of child-parent relationship, the process of stressful and traumatic experiences, identifying personal strengths, coping techniques. - Self-maintenance tools like breathing, meditation, relaxation, physical exercises, self-affirmation, and guided imagery were taught and practiced. | x | x | - | - | - | - | x | NR | Groups of 15-20 | - | x | - | x | 6-hour sessions for 12 weeks | Attendance: 88.2% |
| Fiol-DeRoque, 2021^13^  CBT-based psychoeducation app | Early evidence found extremely high prevalence of adverse mental health outcomes amongst frontline health workers during COVID-19. | Psychoeducational materials delivered through the PsyCovid App including written and audiovisual content targeting emotional skills, health lifestyle behaviours, work stress and burnout and social support. Daily prompts for self-monitoring, followed by tailored responses were also included. | - | x | - | x | - | x | x | App content developed by psychologists, psychiatrists, and experts in healthy lifestyle promotion | Self-paced | x | - | x | - | Daily prompts, self-paced for 2 weeks | Usability scores 87.21/100; 94.1% asked to regain access following intervention period |
| Gnanapragasam, 2023^14^  Mobile app for mental well-being, stress, sleep | HCW providers faced considerable work-related pressure due to COVID-19 which can affect not only their mental health, but also patient care. | Educational information focused on one of six areas: relaxation, sleep, anxious thoughts, feeling down, self-esteem, stress. Users can self-select content or receive recommendations. Includes a variety of stand-alone features, and ongoing programmes. | - | x | - | - | - | x | x | Previously developed and tested app | Self-paced | x | - | x | - | Encouraged daily use for 2-weeks, then continuing as needed. | NR |
| Karakashian, 1994^15^  Didactic-experiential group seminars, post-earthquake | Intergenerational issues and shared cultural history may affect therapists treating trauma following a mass casualty event. | Didactic seminars in both a structured and an unstructured group process; discussion of coping strategies, issues related to school consultation and treatment of trauma, establishment of an ongoing peer-support group. | x | - | x | x | - | - | - | 25 female school counsellors | Group workshops | - | x | - | x | Single, 2.5h session | NR |
| Leitch, 2009^16^  Somatic Experience®/ Trauma Resiliency Model™ (SE/TRM) and Social Service Workers following Hurricanes Katrina and Rita | In disasters, social service workers are often survivors themselves. The traumatic stress reactions that often follow can hinder the ability of local caregivers to function at pre-disaster levels. | - Group and individual sessions focused on self-regulation, emotions, cognitions, and concrete skills to reduce hyperarousal and dysregulation by tracking nervous system shifts through physical symptoms - Secondary individual sessions focused on self-regulation and identifying associated emotions and cognition. | x | x | - | x | - | - | x | Two SE-trained clinicians from the United States or Canada led each group. The clinicians had a minimum of two years of the three-year SE training. | Workshops | - | x | x | x | 90-min  2x40-60 min 1:1 sessions over 1-2wks | NR |
| Mahaffey, 2021^17^  Disaster worker resiliency training workshop following Hurricane Sandy | HCW are often exposed to traumatic psychological experiences during disaster response and are at increased risk for physical and mental health problems. | HCW participated in small group training. They received a training manual, an instructor training manual and a digital presentation covering natural, and human caused disasters, stress and resilience, chronic stress, and PTSD. Participants identified factors for resiliency, the importance of social support and asking for help. Participants practiced motivational interviewing and relaxation exercises. | x | x | - | - | - | - | x | Trained MH professionals (clinical psychologist or social worker) | Large group experiential learning | - | x | - | x | One session spanning 4 hours | NR |
| Pallavicini, 2022^18^  Virtual reality-based psychoeducation | Virtual reality-based psychoeducation may be more effective by placing users' subjective experience in a more relaxing environment | Immersive virtual reality psychoeducational experience on stress and anxiety, set in a naturalistic relaxing setting (tropical island). Consisted of three educational "paths" 1) definitions of stress and anxiety; 2) causes and symptoms of anxiety; 3) main treatment of anxiety | - | x | - | - | - | - | x | Researcher developed using user-centred design approaches | Virtual reality session | x | - | x | - | Single session | NR |
| Powell, 2016^19^  Resilience and Coping for the Healthcare Community intervention (RCHC) following Hurricane Sandy | HCW in disaster affected communities play a dual role of survivor and caregiver. Post-disaster emotional reactions include job burnout, compassion fatigue, secondary traumatic stress, vicarious traumatization, and depression. | RCHC program provides information on post-disaster stress reactions, care provider reactions to stress and trauma and coping strategies through interactive psychoeducation. Practical approaches are provided to expand the capacities of health care and social service professionals to support patients, colleagues, themselves, and their families. There is a solution-focused approach used to strengthen self-efficacy around coping strategies and to help participants take a proactive role in amplifying individual, familial and community strengths and resources. | x | - | - | - | - | - | - | NR | Single, group-based workshop | - | x | - | x | Single, 3-h workshop | NR |
| Powell, 2019^20^  Caregivers Journey of Hope (CJoH) stress reduction program following Hurricane Sandy | HCW are at a high risk of experiencing disaster-related distress. Many are providing care to disaster affected individuals while also directly experienced the event. | Group-based stress reduction program   - Introduction: establish trust and setting the rules - Part 1: Identifying types, sources of stress; - Part 2: Cognitive adaptations to stress and the impact of stress; - Part 3: Resources and coping mechanisms, self-care and experiential learning; - Part 4: Future visions goals and plans. Small groups brainstorm steps to build a positive future post disaster. | x | - | x | - | - | - | - | Save the Children | Large group setting followed by smaller groups of 3-4 | - | x | - | x | Half-day workshop | NR |
| Yi-Frazier, 2022^21^  Promoting Resilience in Stress Management (PRISM) Resilience coaching manual | Frontline HCW under immense pressure and distress during COVID-19, previous work has found a skills-based stress management program can support HCW | 6-session, manualized, skills-based training program including 1) the science of resilience; 2) stress management; 3) goal setting; 4) cognitive reframing; 5) meaning-making; and 6) coming together and moving forward with resilience | x | x | - | x | - | - | - | PRISM-trained health coaches with masters and doctorate level psychology or social work degrees | Facilitated group sessions | x | - | - | x | 6, 1-hour sessions | 100% delivery fidelity |
| Mind-body interventions (n = 11) | | | | | | | | | | | | | | | | | |
| Hosseinzadeh, 2021^22^  Mindfulness meditation | The COVID-19 pandemic has exacerbated the challenges of vulnerable populations hat social workers serve; this has negative implications on physical and mental health. | Brief, mindfulness-based cognitive training. Each session included Part 1: Mindfulness-based psychoeducational materials.  Part 2: Meditation exercises | x | x | - | x | - | - | x | PhD-level clinical social worker | NR | x | - | NR | NR | 4 x 1x/week, 70-min + 10-20 min daily homework | NR |
| Hsieh, 2022^23^  Gong meditation | Nurses have faced extreme psychological pressure working during the COVID-19 pandemic | A Garmin smartwatch was worn to measures stress. During gong meditation sessions, participants lay on sleeping pads and covered with blankets. During the guided meditation, the gong was struck with a real-time adjusted rhythm. | - | - | - | - | - | - | - | Qualified gong therapist | Structured meditation | - | x | NR | NR | 7 x60 min sessions over 2 days | NR |
| Ibrahim, 2022^24^  Mindfulness breathing via WhatsApp | Nurses have been disproportionately impacted by the COVID-19 pandemic, representing nearly half of the COVID-19 fatalities | A WhatsApp group was created with detailed protocol and video practice guidelines. Participants practised mindfulness breathing practices independently, followed by a reflective form. | - | x | - | - | - | - | x | Researchers via video | Self-led mindfulness breathing practice | x | - | - | x | 2x/week x 4 weeks, 15-min per session | NR |
| Iwakuma, 2017^25^  Breathing based meditation for HCWs post-earthquake | Many evacuees of the Great East Japan Earthquake were HCW who risked their lives to help rescue others. HCWs in the aftermath of disasters are at a high risk for post-traumatic stress disorder. | A breathing-based meditation session was offered while relaxing music was played, followed by a reflection session in which each participant was asked to comment on his or her meditation experiences. | x | - | - | - | - | - | x | A chief priest of the Hida Senkoji temple provided meditation instruction | Structured meditation | - | x | - | x | Single, 45-min session | NR |
| Joshi, 2022^26^  Transcendental meditation | HCW are experiencing high levels of burnout and stress because of the COVID-19 pandemic | Transcendental meditation instruction including review of scientific research, discussion of the methods and origin, personal instruction, verification of correct practice and review of techniques, and discussion of human potential and optimal wellness | x | - | - | x | - | - | x | Certified meditation instructor | Mix of 1:1 training and group-based review of techniques | NR | NR | x | x | 5, 75-min sessions with 3 optional 45-min follow-up sessions | 92.7% adherence to training; 65.9% practiced 2x/day, 26.8% practiced daily |
| Keng, 2022^27^  Headspace meditation app | HCW are at greater risk of psychological distress than the general population during the COVID-19 pandemic. | Following an introduction to the app, participants were asked to follow Headspace's 10-day basic course, consisting of daily 10-min practices including mindful breathing, mindfulness of thoughts, mindfulness of sounds. | - | x | - | - | - | - | x | Headspace app | Self-paced | x | - | x | - | 10 sessions over 21 days | Mean total of 185.31 (SD: 88.65) min of practice; 88.2% completion rate |
| Li, 2022^28^  Brief mindfulness meditation | The COVID-19 pandemic has placed undue stress on HCW, with surveys showing high prevalence of depression, anxiety, insomnia, and distress | Following an introductory lecture, participants followed a brief mindfulness meditation practice | - | - | - | - | - | - | x | NR | Independently practiced, synchronously with other participants | x | - | - | x | Daily 15-min practice for 16 days | NR |
| Liu, 2015^29^  Ba Duan Jin for International HCW | International HCW perform medical tasks in unfamiliar countries and face patients with complex and critical illnesses while lacking social support. This may lead to a serious deterioration in QoL. | Team members received a 1x/week training of Ba Duan Jin followed by daily practice in comfortable clothes. | x | - | - | - | - | - | x | NR | Workshop plus daily individual practice | - | x | x | x | Single workshop  Home practice 30-m/day for 6wks | NR |
| Si, 2022^30^  Laughter yoga | To work efficiently during the COVID-19 pandemic, nurses need to feel physically and mentally well; laughter yoga can contribute to a more positive environment | Laughter yoga including deep breathing exercises to prepare the lungs for laughter (5 min), warm-up exercises (stretching, songs, clapping, body movements, 10 min), childish games (10 min), laughter exercises (15 min). | - | x | - | - | - | - | x | NR | Minimum of 5 nurses per group; average ~ 20 | x | - | - | x | 2x/week for 8 weeks |  |
| Waelde, 2008^31^  Mindfulness and mantra training (Inner Resources for Stress) to reduce PTSD in HCW following Hurricane Katrina | Post-disaster interventions should address hyper-arousal, which may be key in the development of PTSD and may increase over time following disasters. | Primary intervention included mindful awareness of breath; breath-focused imagery; and letting go, which involves observing thoughts, feelings, and experiences as they arise. A home study program used a program manual, participants performed weekly mindfulness practice. | x | x | - | - | - | - | x | Mindfulness instructor | Workshop plus home practice | - | x | x | x | Single, 4h workshop  10 weeks home study | NR |
| Waelde, 2018^32^  Mindfulness and mantra training (Inner Resources for Stress) for disaster mental HCW following Typhoon Haiyan | There is growing recognition that providers themselves may be disaster-exposed, prompting attention to their stress symptoms and needs for self-care. | Primary intervention included mindful awareness of breath; breath-focused imagery; and letting go, which involves observing thoughts, feelings, and experiences as they arise. A home study program used a program manual, participants performed weekly mindfulness practice. | x | x | - | - | - | - | x | Mindfulness instructor | Workshop plus home practice | - | x | x | x | Single, 4h workshop  30-min/day, 6da/week for 8 weeks home study | 24.4% completed program; mean meditation time = 481.5 +/- 103 min |
| Yildirim, 2022^33^  Mindfulness-based music therapy | Nurses providing care to COVID-19 patients face excessive stress, and work-related strain which can adversely impact wellbeing. | Information was presented on the impact of breathing on the body and mind. Breathing exercises were then performed while light piano music was played. | - | x | - | x | - | - | x | Certified therapist | Single, guided group session | x | - | - | x | Single, 30-min session | NR |
| Organizational interventions (n = 3) | | | | | | | | | | | | | | | | | |
| Chen, 2005^34^  In-service training & MH support during SARS | SARS manifests as an intra-hospital infection leaving HCW anxious and fearful. | - Coordination of management team’s work and publicity - Formulation of the psychological intervention materials, rules, guidance & supervision - Clinical psychological intervention for HCWs and patients - Telephone hotline team for MH problems | - | x | - | x | - | - | - | Psychiatrists, social workers, psychological counselors, and psychiatric nurses | Workplace policies, resources, interpersonal support, and telephone guidance | x | x | x | - | N/A | NR |
| Saqib, 2020^35^  Staff well-being hub during COVID-19 | Social distancing measures are impacting the staff’s ability to destress and cope with usual family and friend support. | A well-being hub with relaxed furnishing and décor; board games, mindfulness activities and books; donated paintings; yoga and mindfulness sessions. Staff enter through their identity badge. A set of rules has been displayed, requesting handwashing and social distancing. | - | x | x | - | - | - | x | Various staff members volunteered their time to lead yoga and mindfulness sessions for staff. | Wellness hub self-directed  interpersonal activities | - | x | x | x | N/A | NR |
| Zaghini, 2021^36^  Management, care reorganization, staffing, education, surveillance | Health care administrators recognized the importance of proactive action to prevent adverse mental health effects. | Nurses’ environment: reorganized care, created new wards, developed new protocols, enhanced isolation  Staff: increased from 1:9 to 1:6 in medium care intensity and 1:4 to 1:2 in high intensity units;  Competence and learning related to SARS-CoV-2 and IPAC measures  Psychological supports  Enhanced surveillance of nurses and staff | - | x | - | - | - | - | - | Healthcare administrators | Organizational restructuring | - | x | x | x | N/A | NR |
| Other interventions (n = 2) | | | | | | | | | | | | | | | | | |
| Giordano, 2020^37^  Music therapy playlists for HCWs during COVID-19 | The COVID-19 situation in Northern Italy had a significant impact on the physical and mental wellbeing of HCW who are under increasing daily pressure of being infected and of infecting family members, and the stress associated with the loss of patients, colleagues loved ones. | Specific playlists were created for relaxation and to reduce anxiety and stress (“Breathing”, classical music), to recover energy and support concentration (“Energy”, pop, rock, jazz), to release tension and instill calm (“Serenity”). Tracks selected based on pulse, mood, melodic lines; dynamic change; bass line; stability in volume, timbre, rhythm, harmony and pitch. | - | - | - | - | - | - | x | 2 trained music therapists | Delivered to participant’s mobile phones | x | - | x | - | Playlist length was 15-20 minutes; received weekly for 4 weeks | NR |
| Mahdood, 2022^38^  Aromatherapy | Operating room staff have impaired sleep, which has been exacerbated by increased workload during the COVID-19 pandemic | Participants received natural pure oil of damask rose from Barij Essence Pharmaceutical Co, as Rosa damascena as 3.3 mg citronellol in 100g of product. Participants added two drops to a cotton ball, and inhaled 5 cm from nose before shift, and five drops on an absorbent cloth napkin 20cm from nose during sleep. | - | - | - | - | - | - | x | NR | Self-led | NA | NA | NA | NA | On shift days and nightly for 30 nights | NR |
| Legend: HCW: health care worker; MH: mental health; PTSD: post-traumatic psychiatric disorder; PT: physiotherapy; PFA: psychological first aid; NHS: National Health Service; CBT: cognitive behaviour therapy; OT: occupational therapy; AFSP: American Foundation for Suicide Prevention; ICU: intensive care unit; IPAC: infection protection and control; EMS: emergency medical services; PTSD: post-traumatic stress disorder; PFA: psychological first aid; SNF: skilled nursing staff; ICU: intensive care units; QoL: quality of life; LTC: long term care; IR: Inner Resources; ADP: anticipate, plan and deter; NR: not reported, VRE: virtual reality environment | | | | | | | | | | | | | | | | | |

**Supplementary Table 2: Critical appraisal of included studies**

| **Randomized control trials (n = 15)** | | | | | | | | | | | | | | | | | | | | | | | |
| --- | --- | --- | --- | --- | --- | --- | --- | --- | --- | --- | --- | --- | --- | --- | --- | --- | --- | --- | --- | --- | --- | --- | --- |
| Author name, Year | **Randomization** | | **Allocation concealment** | | **Similarity of groups** | | **Blinding of participants to treatment** | | **Blinding of administrators to treatment** | | **Blinding of assessors to treatment** | **Similarly of treatment** | | **Completeness of follow up** | | **Analysis according to randomization** | | **Reliability of outcome measurement** | | **Analysis** | **Appropriate design and analysis** | | **Number of domains rated as unclear or high risk of bias** |
| Berger, 2011^12^ | Yes | | Yes | | Yes | | No | | Unclear | | No | Yes | | Unclear | | Yes | | Yes | | Yes | Yes | | 4 |
| De Kock, 2022^4^ | Yes | | Yes | | Yes | | No | | Yes | | No | Yes | | No | | Yes | | Yes | | Yes | Yes | | 3 |
| Difede, 2007^5^ | Yes | | Yes | | Yes | | No | | No | | No | Yes | | Yes | | Yes | | Yes | | Yes | Yes | | 3 |
| Fiol-DeRoque, 2021^13^ | Yes | | Yes | | Yes | | Yes | | Yes | | Yes | Yes | | Yes | | Yes | | Yes | | Yes | Yes | | 0 |
| Gnanapragasam, 2023^14^ | Yes | | Yes | | Yes | | No | | Yes | | No | Yes | | No | | Yes | | Yes | | Yes | Yes | | 3 |
| Hosseinzadeh, 2021^22^ | Unclear | | Unclear | | Yes | | No | | No | | No | Yes | | No | | Yes | | Yes | | Yes | Yes | | 6 |
| Hsieh, 2022^23^ | Yes | | Unclear | | No | | Unclear | | Unclear | | Unclear | Yes | | Yes | | Yes | | Yes | | Yes | Yes | | 5 |
| Joshi, 2022^26^ | Yes | | Unclear | | Yes | | Unclear | | Unclear | | Unclear | Yes | | Yes | | Yes | | Yes | | Yes | Yes | | 4 |
| Keng, 2022^27^ | Yes | | Unclear | | Yes | | No | | Yes | | No | Yes | | Yes | | Yes | | Yes | | No | Yes | | 4 |
| Li, 2022^28^ | Unclear | | Unclear | | Unclear | | No | | Unclear | | Unclear | Yes | | No | | Yes | | Yes | | No | Yes | | 8 |
| Mahaffey, 2021^17^ | Yes | | Yes | | Unclear | | No | | No | | No | Yes | | No | | No | | Yes | | No | No | | 8 |
| Mahdood, 2022^38^ | Yes | | No | | Yes | | No | | No | | No | Yes | | Yes | | Yes | | Yes | | Yes | Yes | | 4 |
| Si, 2022^30^ | Yes | | Unclear | | Yes | | No | | Unclear | | Unclear | No | | No | | Yes | | Yes | | No | Yes | | 7 |
| Yildirim, 2022^33^ | Yes | | Unclear | | Yes | | Unclear | | Unclear | | Unclear | Yes | | Yes | | Yes | | Yes | | No | Yes | | 5 |
| Zhou, 2022^11^ | Unclear | | Unclear | | No | | No | | Yes | | Yes | No | | Unclear | | Yes | | Yes | | No | Yes | | 7 |
| **Quasi experimental (n = 21)** | | | | | | | |  | | | | | |  | | | | |  | | | | |
| Author name, Year | | **Cause and effect variables** | | **Similarity of study groups** | | **Treatment outside exposure and intervention** | | **Control Group** | | **Multiple outcome measurements** | | | **Completeness of follow up** | | **Consistency of outcome measurement** | | **Reliability of outcome measurement** | | | **Analysis** | | **Number of domains rated as unclear or high risk of bias** | |
| Chen, 2006^34^ | | Yes | | Yes | | Yes | | No | | Yes | | | Yes | | Yes | | Yes | | | Yes | | 1 | |
| Cole, 2021^1-3^ | | Yes | | Yes | | Yes | | No | | Yes | | | No | | Yes | | Yes | | | Yes | | 2 | |
| Giordano, 2020^37^ | | Yes | | Yes | | No | | No | | Yes | | | Unclear | | Yes | | Unclear | | | Yes | | 4 | |
| Ibrahim, 2022^24^ | | Yes | | No | | Unclear | | Yes | | Yes | | | Yes | | Yes | | Yes | | | No | | 3 | |
| Iwakuma , 2017^25^ | | Yes | | Unclear | | No | | No | | No | | | No | | Unclear | | Yes | | | Yes | | 6 | |
| Jing, 2022^6^ | | Yes | | Yes | | Yes | | No | | Yes | | | Yes | | Yes | | Yes | | | Yes | | 1 | |
| Karakashian, 1994^15^ | | Yes | | No | | No | | No | | No | | | Yes | | No | | No | | | Unclear | | 7 | |
| Ke, 2017^7^ | | Yes | | Yes | | No | | No | | No | | | Yes | | Yes | | No | | | No | | 5 | |
| Klomp, 2020^8^ | | Yes | | Unclear | | Unclear | | No | | Yes | | | Unclear | | Yes | | Yes | | | Unclear | | 5 | |
| Leitch, 2009^16^ | | Yes | | No | | Unclear | | Yes | | No | | | Unclear | | Yes | | Yes | | | Yes | | 4 | |
| Leiva-Bianchi, 2018^9^ | | Yes | | No | | No | | Yes | | No | | | Yes | | Yes | | Yes | | | Yes | | 3 | |
| Liu, 2015^29^ | | Yes | | Unclear | | Unclear | | Yes | | No | | | Yes | | Yes | | Yes | | | Unclear | | 4 | |
| Osman, 2021^10^ | | Yes | | Yes | | Yes | | No | | Yes | | | Unclear | | Yes | | Yes | | | Yes | | 2 | |
| Pallavicini, 2022^18^ | | Yes | | Yes | | Yes | | No | | Yes | | | Yes | | Yes | | Unclear | | | Yes | | 2 | |
| Powell, 2016^19^ | | Yes | | Yes | | Yes | | No | | No | | | No | | Yes | | Yes | | | Yes | | 3 | |
| Powell, 2019^20^ | | Yes | | Yes | | Yes | | No | | No | | | Yes | | Yes | | Yes | | | Yes | | 2 | |
| Saqib, 2020^35^ | | Yes | | Yes | | Yes | | No | | No | | | No | | Yes | | No | | | Yes | | 4 | |
| Waelde, 2008^31^ | | Yes | | Yes | | Yes | | No | | No | | | No | | Yes | | Yes | | | Yes | | 3 | |
| Waelde, 2018^32^ | | Yes | | Yes | | Yes | | No | | No | | | No | | Yes | | Yes | | | Yes | | 3 | |
| Yi-Frazier, 2022^21^ | | Yes | | Yes | | Yes | | No | | Yes | | | Yes | | Yes | | Yes | | | Yes | | 1 | |
| Zaghini, 2021^36^ | | Yes | | Yes | | Yes | | No | | Yes | | | Yes | | Yes | | Yes | | | Yes | | 1 | |

**REFERENCES**

1. Cole CL, Waterman S, Hunter ECM, et al. Effectiveness of small group cognitive behavioural therapy for anxiety and depression in Ebola treatment centre staff in Sierra Leone. *Int Rev Psychiatry*. Feb-Mar 2021;33(1-2):189-197. doi:10.1080/09540261.2020.1750800

2. Waterman S, Cole CL, Greenberg N, Rubin GJ, Beck A. A qualitative study assessing the feasibility of implementing a group cognitive-behavioural therapy-based intervention in Sierra Leone. *BJPsych Int*. May 2019;16(2):31-34. doi:10.1192/bji.2018.7

3. Waterman S, Hunter ECM, Cole CL, et al. Training peers to treat Ebola centre workers with anxiety and depression in Sierra Leone. *Int J Soc Psychiatry*. Mar 2018;64(2):156-165. doi:10.1177/0020764017752021

4. De Kock JH, Latham HA, Cowden RG, et al. Brief Digital Interventions to Support the Psychological Well-being of NHS Staff During the COVID-19 Pandemic: 3-Arm Pilot Randomized Controlled Trial. Journal article. *JMIR Ment Health*. Apr 4 2022;9(4):e34002. doi:10.2196/34002

5. Difede J, Malta LS, Best S, et al. A randomized controlled clinical treatment trial for World Trade Center attack-related PTSD in disaster workers. Comparative Study; Journal Article; Randomized Controlled Trial; Research Support, N.I.H., Extramural. *J Nerv Ment Dis*. Oct 2007;195(10):861-5. doi:10.1097/NMD.0b013e3181568612

6. Jing H, Zhang L, Liu Y, et al. Effect of a group-based acceptance and commitment therapy program on the mental health of clinical nurses during the COVID-19 sporadic outbreak period. Journal article. *Journal of nursing management*. 2022;doi:10.1111/jonm.13696

7. Ke YT, Chen HC, Lin CH, et al. Posttraumatic Psychiatric Disorders and Resilience in Healthcare Providers following a Disastrous Earthquake: An Interventional Study in Taiwan. *Biomed Res Int*. 2017;2017:2981624. 2981624. doi:10.1155/2017/2981624

8. Klomp RW, Jones L, Watanabe E, Thompson WW. CDC's Multiple Approaches to Safeguard the Health, Safety, and Resilience of Ebola Responders. *Prehosp Disaster Med*. Feb 2020;35(1):69-75. doi:10.1017/S1049023X19005144

9. Leiva-Bianchi M, Cornejo F, Fresno A, Rojas C, Serrano C. Effectiveness of cognitive-behavioural therapy for post-disaster distress in post-traumatic stress symptoms after Chilean earthquake and tsunami. In Press. *Gac Sanit*. May - Jun 2018;32(3):291-296. doi:10.1016/j.gaceta.2017.07.018

10. Osman I, Hamid S, Singaram VS. Efficacy of a brief online mindfulness-based intervention on the psychological well-being of health care professionals and trainees during the COVID-19 pandemic: A mixed method design. *Health SA*. 2021;26:1682. a1682. doi:10.4102/hsag.v26i0.1682

11. Zhou K, Kong J, Wan Y, et al. Positive impacts of e-aid cognitive behavioural therapy on the sleep quality and mood of nurses on site during the COVID-19 pandemic. Journal article. *Sleep Breath*. Dec 2022;26(4):1947-1951. doi:10.1007/s11325-021-02547-1

12. Berger R, Gelkopf M. An intervention for reducing secondary traumatization and improving professional self-efficacy in well baby clinic nurses following war and terror: a random control group trial. *Int J Nurs Stud*. May 2011;48(5):601-10. doi:10.1016/j.ijnurstu.2010.09.007

13. Fiol-DeRoque MA, Serrano-Ripoll MJ, Jimenez R, et al. A Mobile Phone-Based Intervention to Reduce Mental Health Problems in Health Care Workers During the COVID-19 Pandemic (PsyCovidApp): Randomized Controlled Trial. Journal Article; Clinical Trial Protocol. *JMIR Mhealth Uhealth*. May 18 2021;9(5):e27039. doi:10.2196/27039

14. Gnanapragasam SN, Tinch-Taylor R, Scott HR, et al. Multicentre, England-wide randomised controlled trial of the 'Foundations' smartphone application in improving mental health and well-being in a healthcare worker population. Trial registry record; Clinical trial protocol. *Br J Psychiatry*. Feb 2023;222(2):58-66. doi:10.1192/bjp.2022.103

15. Karakashian M. Countertransference issues in crisis work with natural disaster victims. *Psychotherapy: Theory, Research, Practice, Training*. Sum 1994;31(2):334-341.

16. Leitch ML, Vanslyke J, Allen M. Somatic experiencing treatment with social service workers following Hurricanes Katrina and Rita. *Soc Work*. Jan 2009;54(1):9-18. doi:10.1093/sw/54.1.9

17. Mahaffey BL, Mackin DM, Rosen J, Schwartz RM, Taioli E, Gonzalez A. The disaster worker resiliency training program: a randomized clinical trial. Journal: Article in Press. *Int Arch Occup Environ Health*. Jan 2021;94(1):9-21. doi:10.1007/s00420-020-01552-3

18. Pallavicini F, Orena E, i Santo S, et al. A virtual reality home-based training for the management of stress and anxiety among healthcare workers during the COVID-19 pandemic: study protocol for a randomized controlled trial. Journal Article; Clinical Trial Protocol. *Trials*. 2022;23(1)

19. Powell T, Yuma-Guerrero P. Supporting Community Health Workers After a Disaster: Findings From a Mixed-Methods Pilot Evaluation Study of a Psychoeducational Intervention. *Disaster Med Public Health Prep*. Oct 2016;10(5):754-761. doi:10.1017/dmp.2016.40

20. Powell TM, Wegmann KM, Shin OJ. Stress and coping in social service providers after Superstorm Sandy: An examination of a postdisaster psychoeducational intervention. *Traumatology*. June 2019;25(2):96-103. doi:10.1037/trm0000189

21. Yi-Frazier JP, O'Donnell MB, Adhikari EA, et al. Assessment of Resilience Training for Hospital Employees in the Era of COVID-19. *JAMA Netw Open*. Jul 1 2022;5(7):e2220677. e2220677. doi:10.1001/jamanetworkopen.2022.20677

22. Hosseinzadeh A, Navid R. A randomized controlled trial of a mindfulness-based intervention in social workers working during the COVID-19 crisis. Article. *Current Psychology*. 2021:1-8.

23. Hsieh HF, Huang YT, Ma SC, Wang YW. Occupational Burnout and Stress of Nurses in Taiwan Regarding COVID-19: an Intervention with Gong Medication. Journal article. *Journal of nursing management*. 2022;doi:10.1111/jonm.13653

24. Ibrahim K, Komariah M, Herliani YK. The Effect of Mindfulness Breathing Meditation on Psychological Well-being: a Quasi-Experimental Study Among Nurses Working for COVID-19 Patients. Journal article. *Holistic nursing practice*. 2022;36(1):46‐51. doi:10.1097/HNP.0000000000000464

25. Iwakuma M, Oshita D, Yamamoto A, Urushibara-Miyachi Y. Effects of Breathing-Based Meditation on Earthquake-Affected Health Professionals. *Holist Nurs Pract*. May/Jun 2017;31(3):177-182. doi:10.1097/HNP.0000000000000211

26. Joshi S, Vaishnavi S, Brucker A, et al. Targeting Healthcare Provider Burnout During the COVID-19 Pandemic. Journal article; Conference proceeding. *American journal of respiratory and critical care medicine*. 2022;205(1)doi:10.1164/ajrccm-conference.2022.205.1_MeetingAbstracts.A3690

27. Keng SL, Chin JWE, Mammadova M, Teo I. Effects of Mobile App-Based Mindfulness Practice on Healthcare Workers: a Randomized Active Controlled Trial. *Mindfulness (N Y)*. 2022;13(11):2691-2704. doi:10.1007/s12671-022-01975-8

28. Li JM, Wu R, Zhang T, et al. Psychological responses of medical staff during COVID-19 and the adjustment effect of brief mindfulness meditation. *Complement Ther Clin Pract*. Aug 2022;48:101600. doi:10.1016/j.ctcp.2022.101600

29. Liu B, Zhu Y, Huang S. Influence of TCM Baduanjing exercise on physical and mental condition of international medical team members fighting against Ebola virus. (English). *Chinese Nursing Research*. 2015;29(7C):2629.

30. Si SCKA, Kilinc T. The effect of laughter yoga on perceived stress, burnout, and life satisfaction in nurses during the pandemic: a randomized controlled trial. Journal article. *Complementary therapies in clinical practice*. 2022;49:101637‐.

31. Waelde LC, Uddo M, Marquett R, et al. A pilot study of meditation for mental health workers following Hurricane Katrina. *J Trauma Stress*. Oct 2008;21(5):497-500. doi:10.1002/jts.20365

32. Waelde LC, Hechanova M, Ramos PAP, Macia KS, Moschetto JM. Mindfulness and mantra training for disaster mental health workers in the Philippines. *Mindfulness*. Aug 2018;9(4):1181-1190.

33. Yildirim D, Ciris Yildiz C. The Effect of Mindfulness-Based Breathing and Music Therapy Practice on Nurses' Stress, Work-Related Strain, and Psychological Well-being During the COVID-19 Pandemic: A Randomized Controlled Trial. Journal article. *Holist Nurs Pract*. May-Jun 01 2022;36(3):156-165. doi:10.1097/HNP.0000000000000511

34. Chen R, Chou KR, Huang YJ, Wang TS, Liu SY, Ho LY. Effects of a SARS prevention programme in Taiwan on nursing staff's anxiety, depression and sleep quality: a longitudinal survey. *Int J Nurs Stud*. Feb 2006;43(2):215-25. doi:10.1016/j.ijnurstu.2005.03.006

35. Saqib A, Rampal T. Quality improvement report: setting up a staff well-being hub through continuous engagement. *BMJ Open Qual*. Aug 2020;9(3)doi:10.1136/bmjoq-2020-001008

36. Zaghini F, Fiorini J, Livigni L, Carrabs G, Sili A. A mixed methods study of an organization's approach to the COVID-19 health care crisis. *Nurs Outlook*. Sep-Oct 2021;69(5):793-804. doi:10.1016/j.outlook.2021.05.008

37. Giordano F, Scarlata E, Baroni M, et al. Receptive music therapy to reduce stress and improve wellbeing in Italian clinical staff involved in COVID-19 pandemic: A preliminary study. *Arts Psychother*. Sep 2020;70:101688. doi:10.1016/j.aip.2020.101688

38. Mahdood B, Imani B, Khazaei S. Effects of Inhalation Aromatherapy With Rosa damascena (Damask Rose) on the State Anxiety and Sleep Quality of Operating Room Personnel During the COVID-19 Pandemic: A Randomized Controlled Trial. Journal article. *J Perianesth Nurs*. Aug 2022;37(4):493-500. doi:10.1016/j.jopan.2021.09.011
